# Nitrogen Fixation Associated with *Microcystis* Colonies Promotes Harmful Cyanobacterial Blooms across North American Lakes

**DOI:** 10.1021/acs.est.5c13196

**Published:** 2026-01-22

**Authors:** Christopher J. Gobler, Ann Marie E. Famularo-Pecora, Benjamin J. Kramer, Jennifer G. Jankowiak, Jennifer A. Goleski, Ronojoy Hem, Kendra A. Turk-Kubo, Jonathan P. Zehr

**Affiliations:** † School of Marine and Atmospheric Sciences, 52391 Stony Brook University, Southampton, New York 11968, United States; ‡ USGS Great Lakes Science Center, Ann Arbor, Michigan 48105, United States; § Ocean Sciences Department, 118562 University of California, Santa Cruz, California 95064, United States

**Keywords:** Microcystis, cyanobacterial harmful algal blooms, N_2_ fixation, nifH

## Abstract

*Microcystis* forms harmful cyanobacterial algal blooms around the world, including regions with low inorganic nitrogen (N) concentrations. Here, we measured the N_2_ fixation rates of isolated *Microcystis* colonies, free-living plankton, and the whole plankton community during dense *Microcystis* blooms over a three year period in six diverse lakes across eastern North America including Lake Erie. For five of six lakes, rates within the colony fraction were greater than the rates measured for free-living plankton (*p* < 0.05). N_2_ fixation rates were inversely correlated with the rates of ammonium uptake by the colony fraction (*p* < 0.05), and in experiments, ammonium significantly reduced N_2_ fixation rates within the colony fraction by 50–85% (*p* < 0.001). In several lakes, the δ^15^N of isolated colonies was lower (*p* < 0.05) than that of free-living plankton presumably due to the preferential use of ^14^N_2_ by diazotrophic, colony-associated plankton. Cyanobacterial and noncyanobacterial *nifH* sequences were associated with the *Microcystis* colonies with the *nifH*-based diversity of both groups being lower in colonies compared to free-living plankton. Compared to nitrate, ammonium, and urea uptake, N_2_ fixation rates accounted for 1–76% of total N assimilation by the *Microcystis* colony fraction and averaged 18% across systems, suggesting this process could support the proliferation of *Microcystis* blooms.

## Graphical abstract

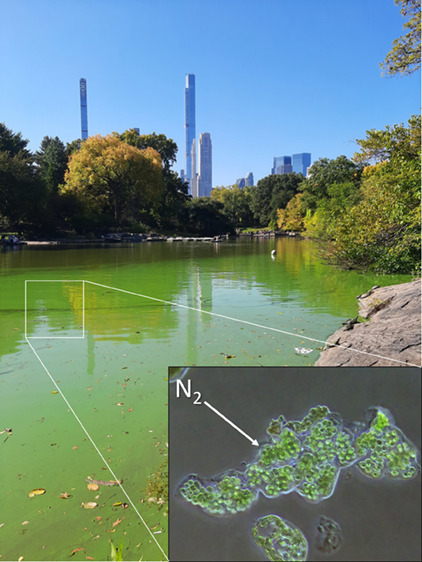

## 1 Introduction

Harmful algal blooms (HABs) are a complex and expanding threat to coastal zones across the globe.^1^ Within freshwater ecosystems, cyanobacterial harmful algal blooms (CHABs) can produce potent toxins that can threaten human and animal health as well as disrupt ecosystems and economies.^2^ CHABs are known to be promoted by warmer temperatures and excessive nutrient loading.^3–5^ While primary productivity in freshwater ecosystems has traditionally been viewed as strictly phosphorus (P) limited,^6^ the role of nitrogen (N) in promoting both the intensity and toxicity of CHABs has been increasingly recognized in recent decades.^3,7,8^

Globally, *Microcystis* is the most common CHAB genus and the toxin it synthesizes, microcystin, is commonly regulated in drinking water and recreational waters.^9,10^ As a nondiazotrophic or nondinitrogen (N_2_)-fixing genus, *Microcystis* rely on bioavailable, fixed N (nitrate, ammonium) to form CHABs, and many studies have noted the ability of N to control the growth and microcystin content of this genus.^3,10,11^ Paradoxically, blooms of *Microcystis* can persist for extended periods when levels of dissolved inorganic N are low,^3,7,12,13^ suggesting an ability to access nontraditional forms or pools of N and/or superior N assimilation rates compared to competing plankton.

*Microcystis* is a colony-forming CHAB genus, with colonies characterized as dense aggregations of mucilage inhabited by a diverse collective of prokaryotes.^14–16^ Studies of this *Microcystis* ‘phycosphere’ have demonstrated that the composition of this endosymbiotic bacterial community is significantly different from ambient, pelagic (i.e., free-living) bacterial communities and is conserved with regard to its composition and biochemical functionality across global ecosystems.^17–20^ Among the functionalities that have been putatively identified within the phycosphere are gene sets associated with nitrogenase with taxa potentially capable of N_2_ fixation being up to 4-fold more abundant within *Microcystis* colonies compared to free-living bacteria.^17^ To date, however, diazotrophs associated with *Microcystis* colonies are yet to be identified, and N_2_ fixation associated with *Microcystis* colonies has not been measured. Here, we studied the dynamics of N utilization, including diazotrophy, by isolated *Microcystis* colonies and other plankton during CHABs in six diverse lakes across eastern North America while concurrently characterizing the bacteria responsible for diazotrophy via high-throughput amplicon sequencing of the *nifH* gene. We hypothesized that the *Microcystis* phycosphere recruits specific diazotrophic assemblages that contribute a substantial proportion of the colony’s N requirements, exceeding the fixation rates of the free-living plankton community.

## 2 Materials and Methods

### 2.1 Sample Sites and Collection

For this project, six lakes across North America were sampled, including Maumee Bay within the western basin of Lake Erie (41.823817, −83.331580), and five lakes across New York State: Lake Agawam (40.88148, −72.39256; 0.5 km^2^), the Lake in Central Park (40.77458, −73.97073; 0.5 km^2^), Lake Neatahwanta (43.31385, −76.42956; 3.1 km^2^), Honeoye Lake (42.75582, −77.50968; 7.2 km^2^), and Lake Chautauqua (42.113318, −79.287103; 53 km^2^), between 2020 and 2022 (Figures S1 and S2). All locations are prone to dense blooms of *Microcystis.* ^8,17,21^ Lake Erie is the smallest and shallowest of the North American Great Lakes and the largest of the systems studied (∼2.5 × 10^4^ km^2^). While Lake Neatahwanta, Honeoye Lake, and Lake Chautauqua are medium-sized lakes (3, 7, and 53 km^2^, respectively), Lake Agawam and the Lake in Central Park are small lakes (both <0.3 km^2^). From 2020 to 2022, Lake Agawam and the Lake in Central Park were sampled weekly to monthly as a time series during late spring, summer, and early fall, whereas sites in western Lake Erie were sampled in September 2020 and mid-to-late August of 2021 and 2022, Chautauqua Lake was sampled twice in the summer of 2020, and Lake Neatahwanta and Honeoye Lake were sampled once in the summer of 2021. For all sites, surface water samples were collected with a 5 L Van Dorn bottle used to fill 20 L polycarbonate carboys. A YSI model 556 ProQuatro multiparameter sonde was used to measure temperature, dissolved oxygen, and pH, and a bbe Moldaenke FluoroProbe was used to measure in vivo chlorophyll *a* (chl-*a*) and concentrations of cyanobacteria (cyano chl-*a*).^22^ Total chlorophyll *a* was measured using standard fluorometric techniques,^23^ and values of in vivo chlorophyll *a* were adjusted based on a regression with hundreds of extracted chlorophyll *a* measurements. Identification of dominant cyanobacterial genera was performed using an inverted Nikon Eclipse TS100 microscope and a gridded 1 mm^2^ Sedgewick Rafter counting chamber. Additionally, duplicate samples were collected for the analysis of total (whole water) microcystin. Microcystin samples were analyzed by an ABRAXIS Microcystin/Nodularians test kit according to the manufacturer’s (Gold Standard Diagnostics) recommended procedures. Duplicate samples for analysis of total (whole water) and dissolved (filtered through a combusted EMD Millipore APFB glass fiber filter, combusted for 2 h @ 450 °C) nutrients were collected and stored at −20 °C until further processing. Nutrient samples were analyzed for nitrate, ammonium, orthophosphate, and urea on a Lachat Instruments flow injection system (ASX-520 series) using standard wet chemical methods^23,24^ that achieved 96 ± 9% recovery of standard reference material (SPEX CertiPrep). In 2021 and 2022, triplicate samples were obtained for DNA-based community analysis of the cyanobacterial 16S rRNA (16S rRNA) and diazotrophs (*nifH*) by filtering 25–50 mL of whole water, the isolated colony fraction, and free-living plankton (<20 μm; see Section 2.2 for details) on 0.22 μm polycarbonate filters (Millipore). Filters were immediately frozen in liquid N_2_ and stored in −80 °C until extraction (see Section 2.5 for details).

### 2.2 Microcystis Colony Isolation

To examine the characteristics of whole plankton communities, *Microcystis* colonies, and free-living plankton, colonies were isolated from bloom water using an approach described in Jankowiak and Gobler.^17^ Sample water was well-mixed prior to passing 1–4 L (dependent on the cyanobacterial density) of lake water through a 20 μm nylon mesh sieve to capture the *Microcystis* colonies while allowing free-living plankton to pass into the filtrate.^14^ To remove large (>20 μm) non-*Microcystis* particles that were also captured by the filter, isolated colonies were then resuspended in 250 mL of 0.2 μm filtered lake water. After several minutes under direct light (∼50 μEin m^2^ s^–1^), *Microcystis* colonies rose to the surface, while the non-*Microcystis* particles and plankton fell from suspension. The colonies were again skimmed off the surface using a 50 mL serological pipet and resuspended twice more into 250 mL of 0.2 μm filtered lake water within ∼ 10 min to further isolate colonies from noncolony particles and plankton. An aliquot of this fraction was examined via microscopy to confirm there was no overt contamination of large particles or non-*Microcystis* phytoplankton. Particles remaining after the removal of floating *Microcystis* colonies were concentrated on a 20 μm mesh and analyzed fluorometrically; their biomass averaged ∼10% of the total algal biomass of whole water. The colony fraction was volumetrically diluted with 0.2 μm filtered lake water back to the densities present prior to concentrating this fraction on the 20 μm mesh. Across experiments, the colony isolation processes produced a density of colonies that was highly correlated with and within 14% of the density of colonies found in unamended, whole water (Figure S3). Triplicate samples (25–50 mL) of whole water, colonies, and free-living plankton were filtered onto 0.2 μm polycarbonate filters and immediately stored at −80 °C until DNA extraction. In addition, the isolated colony fraction, the free-living fraction, and whole water were subsequently used to measure N_2_ fixation and N uptake rates.

### 2.3 N_2_ Fixation Rate Measurements Using the Acetylene Reduction Assay

To measure N_2_ fixation rates, the acetylene (C_2_H_2_) reduction assay method^25–27^ was used, assuming one molecule of N_2_ is fixed for every four molecules of ethyelene (C_2_H_4_) produced.^25–27^ While this assumption has been experimentally affirmed for cyanobacteria, its applicability to aquatic bacteria is less certain and the ratio may vary with physiological and environmental conditions.^25–27^ Acetylene (C_2_H_2_) was made by reacting 7 g of calcium carbide (Fisher Scientific) with 700 mL of deionized water,^28^ with the resulting gas collected in Supelco Tedlar bags. C_2_H_4_ standards were made by injecting 1% ethylene in N_2_ (Airgas) into 20 mL vials sealed with magnetic screw caps, while 1 mL of C_2_H_2_ was injected into the headspace of the same type of vials containing 15 mL of sample and incubated with the analyte for 3–4 h. A portion of the headspace was then injected into a Trace 1310 Gas Chromatograph coupled to a Triplus 500 GC Headspace Autosampler (Thermo Scientific). Ethylene concentrations in standards and samples were visualized and quantified using the Chromeleon Chromatography Data System (CDS) software (Version 7.3). The amount of N_2_ fixed during the incubation period was extrapolated to 24 h (μmol N_2_-fixed L^–1^ day^–1^).^29^ Over the course of all experiments, N_2_ fixation rates measured in the colony and free-living size fractions across all samples were 98 ± 11% of rates measured within whole water, demonstrating that the colony isolation method maintained the physiological integrity of plankton with respect to N_2_ fixation. In some cases, experiments were performed to assess the extent to which fixed N in the form of ammonium would alter the N_2_ fixation rates. Acetylene reduction incubations were performed with and without the addition of 50 μM ammonium (*n* = 3–6 vessels for each) with rates measured under both conditions after a 3–4 h incubation.

### 2.4 Quantifying ^15^N-Uptake Rates of Dissolved Nutrients

Tracer experiments were conducted with ^15^N-labeled compounds to determine net uptake rates by the whole plankton community, isolated colonies, and free-living plankton (<20 μm). Uptake rates of nitrate, ammonium, and urea were measured using additions of highly enriched (≥98%) ^15^N-nitrate, ^15^N-ammonium, and ^15^N-urea.^30–32^ All fixed nitrogenous nutrient isotope additions were made at <10% of measured ambient concentrations. Incubations with dissolved nutrients were performed in 50 mL of sterile polystyrene Nunc flasks (Fisher Scientific). All vessels were placed in an incubator set to the same light and temperature conditions present at the time of sample collection for 60 min after which water was filtered onto precombusted (2 h @ 450 °C) EMD Millipore APFB glass fiber filters.

Subsamples of the initial water samples (whole water, colony fractions, free-living fraction) were also filtered onto precombusted EMD Millipore APFB glass fiber filters to determine the natural abundance of ^15^N of plankton prior to enrichment. All filters were pelletized in tin discs and analyzed at the U.C. Davis Stable Isotope Facility (Davis, CA) using an Elementar vario Micro Cube elemental analyzer (Elementar Analysensysteme GmbH, Hanau, Germany) interfaced to a PDZ Europa 20–20 isotope ratio mass spectrometer (Sercon Ltd., Cheshire, UK). Uptake rates were calculated using equations from Glibert et al.^33^ Rates were considered net uptake as they were not corrected for the effects of isotope dilution;^33^ however, uptake, release, and subsequent reuptake of compounds during incubations were likely limited for dissolved nutrients given the brief incubation period (60 min^33^). The amount of N assimilation originating from N_2_ fixation by the colony fraction was quantified by dividing the N_2_ fixation rates by the sum of the nitrate, ammonium, and urea uptake rates plus the N_2_ fixation rates. Given that the assumed conversion ratio of 4:1 (C_2_H_4_:N_2_) can vary and depends on environmental and physiological conditions,^25–27^ this uncertainty would propagate to the estimation of the contribution of N_2_ fixation to total N assimilation.

### 2.5 DNA Isolation, Sequencing, and Analysis

In total, samples from 20 sites/dates were sequenced in 2021 and 2022 across five locations, resulting in 60 samples from the three plankton fractions (whole water, colony-associated, free-living). For high-throughput amplicon sequence analysis, DNA samples were first extracted from frozen samples using the DNeasy PowerWater kit (QIAGEN) as per the manufacturer’s instructions. Extracted nucleic acids were suspended in 75 μL of nuclease-free water, and DNA was quantified using a Nanodrop 1000 spectrophotometer. Samples were stored at −80 °C following normalization until polymerase chain reaction (PCR) amplification and amplicon sequencing, which was performed at Molecular Research Laboratories (Shallowater, TX). The 16S rRNA gene was amplified using the 515*F*/806R primer set^34,35^ and used to discriminate between cyanobacterial genera.^36,37^ To characterize diazotrophic community composition, the *nifH* gene was amplified using the IGK3/DVV primer set.^38,39^ Of the several genes belonging to the *nif* cluster, which encode for enzymes involved in N_2_ fixation,^40^ *nifH* is preferred for diazotrophic community characterization as it is more conserved than the other genes.^41^

Identifying barcodes were placed on the forward primers for each sample, after which PCR amplification was performed using the HotStarTaq Plus Master Mix Kit (QIAGEN) with the cycling conditions as follows: 1 cycle at 95 °C for 5 min, 30 cycles at 95 °C for 30 s, 1 cycle at 53 °C for 40 s, 1 cycle at 72 °C for 1 min, and a final elongation step at 72 °C for 10 min. The success of the amplification and the relative intensity of the PCR products were visualized on a 2% agarose gel. Following confirmation of amplification, samples were multiplexed using unique dual indices and then pooled together in equal proportions based on their molecular weights and DNA concentrations. Samples were then purified using calibrated Ampure XP beads (Beckman Coulter Life Sciences) and used to prepare a DNA library for paired end reads (20 K; 2 × 300) with an Illumina MiSeq platform. All sequences have been deposited in Genbank under accession number PRJNA1356834.

16SrRNA and *nifH* sequence data were processed using the “Moving Pictures” pipeline for Quantitative Insights into Microbial Ecology 2 (QIIME 2) version 2021.11.^42^ Briefly, demultiplexed paired-end reads were trimmed of their primers and barcodes using the Cutadapt plugin,^43^ then Dada2 was used to merge paired-end reads and produce a frequency table of exact (100%) amplicon sequence variants (ASV), which have been used to taxonomically classify 16S and *nifH* communities,^44–46^ using default parameters for the identification and filtering of chimeric sequences, denoising, and dereplication.^47^ For the taxonomic identification of the 16S data set, the 99% 16S only rep set FASTA and majority consensus seven-level taxonomy files of the SILVA rRNA (16S SSU) release v138 database^48^ were used. BLAST search results were refined by removing sequences >2000 bp and noncyanobacterial taxa. For taxonomic identification of diazotrophic cyanobacteria, the 2017 version of the *nifH* ARB database produced by the Zehr Laboratory,^49^ modified for the Dada2 pipeline,^50^ was used. FASTA and taxonomy files of this refined database (*n* = 940) were generated in QIIME 2 using the get-ncbi-data method from the RESCRIPt package.^51^ Reference sequences from the 16S and *nifH* data sets were trimmed of their primers, then used to train and test a Naive Bayes classifier using the feature-classifier^52^ and classify-sklearn^53^ plugins, respectively.

Prior to classification, representative *nifH* sequences were aligned in MAFFT (version 7) with reference sequences of the *nifH* gene.^54^ Reference sequences were subsequently imported into BioEdit (version 7.2) to trim reference sequences to the length of the representative sequences amplified with the *nifH* primers, degapped, reimported into QIIME 2, and then used to train and test a Naive Bayes classifier on representative sequences with the same plugins used to develop and classify 16S representative sequences. Representative *nifH* nucleotide sequences were used to create an amino acid database in ARB,^55^ which was then aligned in HMMER (version 3.3.2; http://hmmer.org/) against sequences from the online database Pfam^56^ that belong to the Fer4_NifH (PF00142): a family of 4Fe-4S iron sulfur cluster binding proteins.^41,57^ Aligned representative sequences and those of the Pfam database were then transported back into ARB to create a phylogenetic tree in order to determine whether representative sequences clustered into Groups I–III, which include members that produce functional nitrogenases, or Group IV, which contains nitrogenase paralogs produced by taxa that do not fix N_2_.^58–62^ Subsequently, sequences that clustered into Group IV were not utilized for further analyses. Taxonomic assignments for all data sets were confirmed by identifying the representative sequences with the most abundant ASVs using NCBI BLAST.^63^ The dominant (≥5%) taxonomic groups in the 16S and *nifH* communities were identified to the lowest possible taxonomic level. Sequences with the same taxonomic identifications were grouped as a single taxon, primarily at the genus level.

### 2.6 Statistical Analyses

Statistical analyses were performed in R (Version 4.0.3) and SigmaPlot build 15.1.1.26. Three-way analyses of variance (ANOVA) were used to assess differences in N_2_ fixation rates, where site, date, and fraction were the treatment factors. Shapiro–Wilk and Kolmogorov–Smirnov tests were used to confirm that data passed normality, while Fligner–Killeen tests were used to confirm that the data passed normality and homogeneity of variance (*p* > 0.05). Tukey’s HSD tests were then performed post hoc (*p* < 0.05). A Spearman's rank-order correlation matrix was generated to assess the covariance of environmental parameters.

All statistical analyses on 16S rRNA and *nifH* sequencing data were performed in QIIME 2 v2021.4 ^45^ unless otherwise noted. Significant differences in the alpha diversity metrics (richness, evenness, Shannon Index) between treatments and experiments were assessed using Kruskal–Wallis pairwise tests with Benjamini and Hochberg multiple comparison correction.^64^ To visualize significant differences in the bacterial community structures (beta diversity) between the experiments and treatments, principal coordinate analyses (PCoA) were conducted on the ASV inferred Bray–Curtis dissimilarities, followed by PERMANOVA analysis (permutational multivariate analysis of variance; 999 permutations calculated per test) to identify significantly different groups. To determine whether changes in the microbiome communities impacted *Microcystis* growth, significant correlations between the bacterial alpha and beta diversities and the biological parameters (Fluoroprobe-derived cyanobacteria abundance) were assessed using Mantel tests (999 permutations) and Spearman correlations, respectively.

The relative abundance of noncyanobacterial *nifH-*carrying genera and geographic distances were assessed with Mantel tests. This was performed in R version 4.4.0 with the vegan package 2.6–6.1.^65^ Bray–Curtis distances were also determined for these microbial genera. The R package geosphere 1.5–18, which contains a function for calculating Haversine distances, was used to create a distance matrix from geographic coordinates.^66^ The Spearman option was chosen for the Mantel tests, and the R packages ggplot 3.51 ^67^ and ggpubr 0.6.0 ^68^ were used to create a pairwise scatterplot. Values at each geographic location were averaged for better visualization. Mantel tests were also used to determine whether distinct geographic regions (small, hypereutrophic lakes vs larger, eutrophic lakes) as well as the fractions of *nifH*-** carrying noncyanobacteria (colonial or free-living) were significant covariates along with distance affecting dissimilarity in these microbial communities.

## 3 Results and Discussion

Across all lakes studied on all dates, levels of algal biomass were high (chlorophyll *a* = 117 ± 24.5 μg L^–1^ = mean ± SE) with cyanobacteria comprising 87 ± 2.4% of chlorophyll *a* and the isolated colony fraction comprising 88 ± 2.2% of cyanobacterial biomass (Table S1). Among the cyanobacteria, *Microcystis* was the dominant taxa in the colony size fraction in five of the six systems (73 ± 7% of 16S sequences; Figure S4) with the exception being Lake Agawam where the picoplanktonic cyanobacterium *Pseudanabaena* comprised the majority of sequences within the colony fraction (76 ± 5%; Figure S4). *Microcystis* likely dominated cyanobacterial biomass in this system given its ∼50-fold larger cellular biovolume relative to *Pseudanabaena*. Heterotrophic bacterial communities associated within *Microcystis* colonies as determined via 16SrRNA sequencing were significantly different from the surrounding free-living communities (*p* < 0.005; Figure S5A) and also differed across lake systems (*p* < 0.001; Figure S5B), a finding consistent with prior studies.^17–19^

There were measurable levels of N_2_ fixation within the *Microcystis* colony fraction for all sites and, for five of six systems, rates among colonies were significantly greater than the rates measured by free-living plankton (*p* < 0.05; Figure 1). While N_2_ fixation rates were, on average, 3-fold greater in the colony fractions compared to the free-living fraction for the Lake in Central Park, Lake Erie, Honeoye Lake, and Lake Chautauqua, they were only 36% greater in Lake Agawam, the only system where *Microcystis* was not the majority of cyanobacterial sequences within the colony fraction (Figure 1). Across all systems, N_2_ fixation rates by the whole plankton community were highly correlated with the rates of the colony fraction (*p* < 0.0001; Figure S6A), but not with the rates from the free-living plankton, further evidencing the predominance of diazotrophy in the colony fraction. Rates varied across space and time, with the highest N_2_ fixation rates within the *Microcystis* colony fraction quantified in the Lake in Central Park (0.8 μmol N^1–^ L^–1^ h^–1^, August 4, 2021) and the lowest rates found in Lake Agawam (0.01 μmol N^–1^ L^–1^ h^–1^, September 21, 2020; Table S1; Figure S7). For lakes where there were time series measurements of diazotrophy associated with *Microcystis* colonies, N_2_ fixation rates did not display distinct temporal trends from June through October (Figure S7), a period when temperatures (20–28 °C) and nutrient levels (nitrate and ammonium = 4.7 ± 0.9 μM) did not vary dramatically (Table S1). Across all systems, N_2_ fixation rates were significantly correlated with total cyanobacterial biomass (*p* < 0.005; Figure S6B), suggesting that denser blooms created a greater demand for N that was not met by *in situ* fixed N assimilation. Accordingly, rates were generally higher than those measured in systems with lower total cyanobacterial biomass.^69,70^

**Figure 1:**
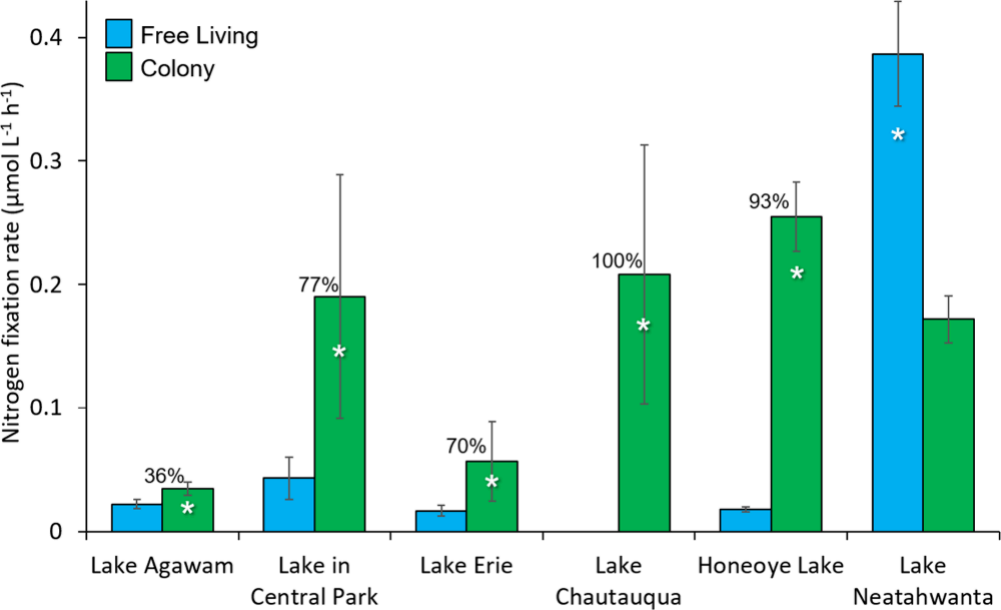
N_2_ fixation rates by the free-living and colony fractions across six lakes. Bars are means with error bars ± 1 SE. Percentages above bars indicate percentage colony-associated rates were greater than the free-living fraction. Asterisks indicate differences were statistically significant (*p* < 0.05).

Ammonium addition (50 μM) experiments, performed in three ecosystems (Lake Agawam, *n* = 5; Lake in Central Park, *n* = 7; Lake Erie, *n* = 1), demonstrated significantly reduced N_2_ fixation rates within the *Microcystis* colony fraction by more than 50% after 4 h (*p* < 0.01 for all; Figure 2 and Table S2). Mean percent reductions in rates were 50, 85, and 60% for Lake Agawam, Lake in Central Park, and Lake Erie, respectively (Figure 2A–C and Table S2; *p* < 0.001). Given the energetic favorability of using ammonium compared to fixing N_2_ when ammonium is available,^71^ these findings indicate that N_2_ fixation associated with colonies was a response to N-limiting conditions and that an excess of ammonium triggered a downregulation of this process. Supporting this concept, across all study sites, the rates of N_2_ fixation by isolated colonies in lakes were inversely and significantly correlated with ammonium uptake rates by colonies (*p* < 0.05), indicating N_2_ fixation by the colony fraction was also downregulated in situ when ammonium levels were elevated.

**Figure 2:**
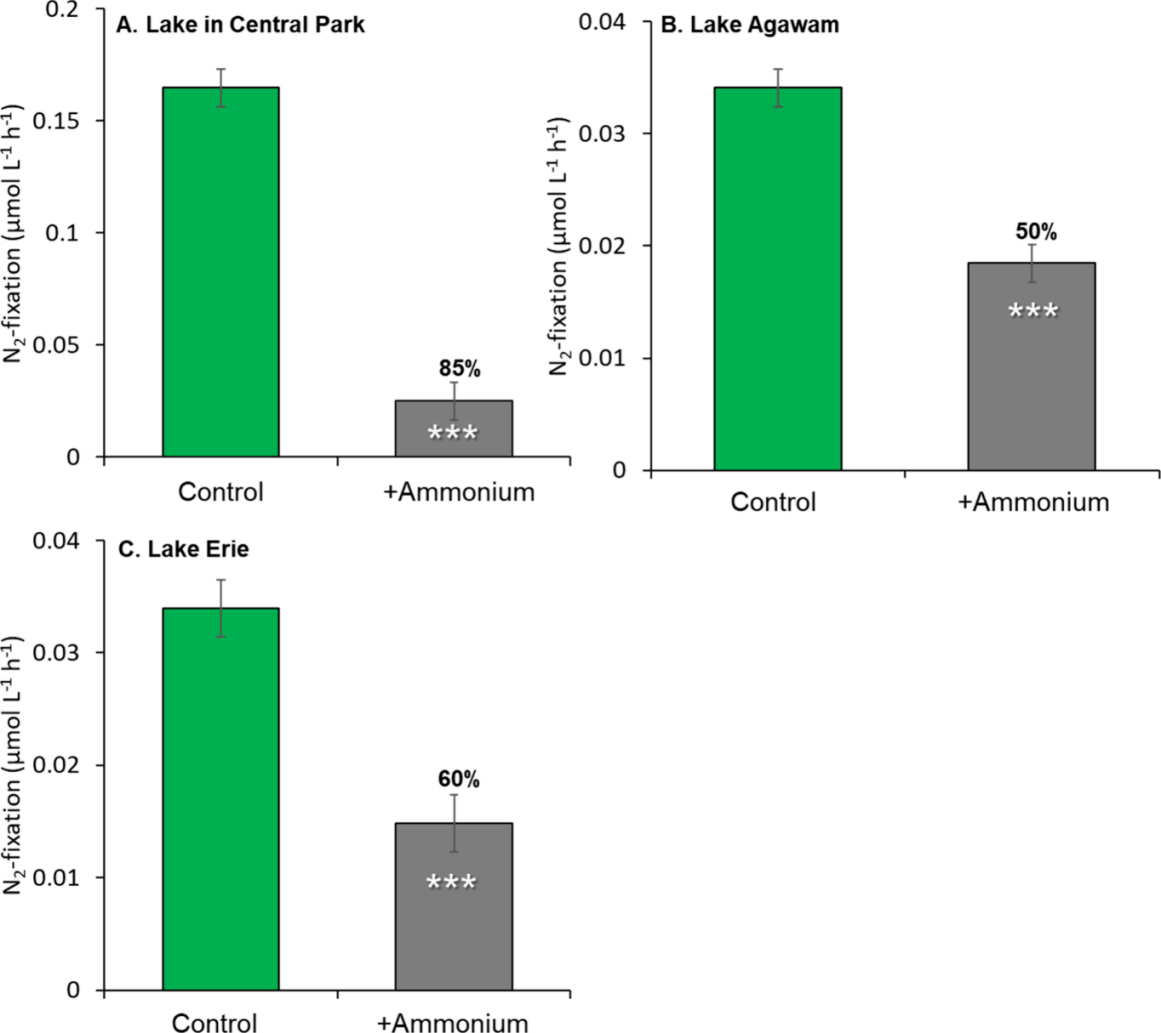
N_2_-fixation by the colony fraction after 4 h with and without ammonium enrichment in (A) Lake in Central Park, 7 experiments; (B) Lake Agawam, 5 experiments; (C) Lake Erie; 1 experiment. Bars are means with error bars ± 1 SE. Percentages above bars indicate percentage rates were reduced. *** indicates differences were statistically significant (*p* < 0.001).

The isotopic signature of nitrogen (δ^15^N) can be used to identify sources of N used by primary producers with lower values being reflective of N_2_ fixation.^72,73^ The existence of N_2_ fixation associated with *Microcystis* colonies was, in some cases, also evidenced by the δ^15^N of isolated colonies compared to free-living plankton within the same system (Figure 3 and Table S3). For example, during two observations of Lake Chautauqua, δ^15^N signatures of isolated colonies (5.7 ± 2.2‰) were significantly lower than free living plankton (18 ± 3.4‰; *p* < 0.05; Figure 3A). Similarly, for Lake Agawam, during 9 of 13 observations, the δ^15^N of the colony size fraction was significantly lower than that of free-living plankton (8.5 ± 0.59 vs 11 ± 2.1‰ *p* < 0.05 for each of the nine comparisons; Figure 3B). Colony fraction δ^15^N was significantly lower than that of free-living plankton twice in Lake Erie and twice in the Lake in Central Park (Figure 3C,D). Values in Lake Erie and Lake Agawam were within the range of prior measurements of δ^15^ N of particulate organic matter in those systems.^74,5^ In some cases, δ^15^N values of all fractions were low (<6‰), and differences between fractions were less pronounced (Table S3). Of the four systems, Lake Chautauqua had the largest difference in δ^15^N between the colony and free-living fractions (>10‰; Figure 3A) and was the only system where N_2_ fixation was undetectable within the free-living plankton during measurements (Figure 1). The lower δ^15^N values of colony-associated plankton are consistent with the isotopic value of a N_2_ gas end member (0 per mil), based on convention.^72,73^ Further, δ^15^N signatures are often used to identify sources of N used by primary producers with lower values (closer to zero) being reflective of N_2_ fixation or fertilizer-derived N and heavier values (above 10) being indicative of wastewater-derived N.^72,73^ We hypothesize that for plankton communities dominated by the use of heavier, wastewater-derived N, N_2_ fixation lowered the δ^15^N in the colony fractions relative to the free-living plankton. This was seemingly the case for Lake Agawam and Lake Chautauqua where δ^15^ N signatures were heavier (≥10‰) than other systems and surrounding municipalities are known to discharge fixed N-rich wastewater to groundwater that seeps into the lakes.^5^

**Figure 3:**
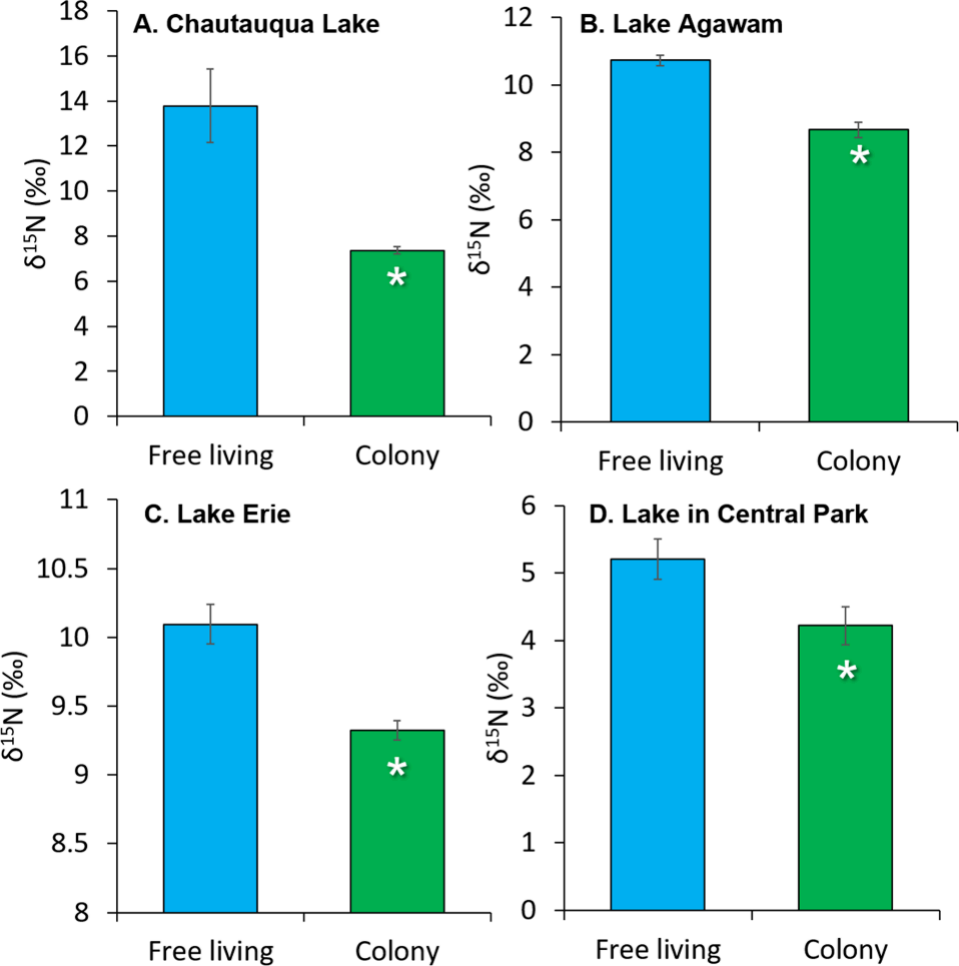
δ^15^N content of free-living plankton and the colony fractions from (A) Chautauqua Lake, *n* = 2 observations; (B) Lake Agawam, *n* = 9 observations; (C) Lake Erie, *n* = 2 observations; (D) Lake in Central Park, *n* = 2 observations. Bars are means with error bars ± 1 SE. Asterisks indicate differences are statistically significant (*p* < 0.05).

High-throughput sequencing of the *nifH* gene within *Microcystis* colonies revealed the presence of sequences of archaeal, noncyanobacterial, and cyanobacterial origin (Figure 4). More than half (51%) of sequences originally identified as *nifH* via standard informatic analyses were found to be Group IV taxa that produce nitrogenase paralogs but do not fix N_2_ ^58–62^ and, therefore, were not considered further. Among noncyanobacterial and archaeal diazotrophs within the colony fraction, *Burkholderia* sp. and *Candidatus methanoregula* were the most prominent *nifH*-containing genera (15% and 11% of sequences, respectively), whereas *Aphanizomenon* sp. and *Dolichospermum* sp. had the greatest relative abundance among the cyanobacterial *nifH* sequences (39 and 31% of sequences, respectively; Figure 4A). These two genera comprised only 4 and 10% of 16S rRNA sequences among colonies across lakes (Figure S4), indicating they were a minor component of biomass, but had an outsized representation among diazotrophs, consistent with other studies of North American lakes.^70,75,76^ While these two cyanobacterial genera form colonies and chains, they commonly exist as single cells and/or single trichomes,^77^ accounting for their greater relative abundance in the free-living fraction (based on 16S for both genera and *nifH* for *Aphanizomenon*) and potentially accounting for their appearance in the colony fraction when not frequently observed microscopically (Figure S8). For both of these cyanobacteria, their ability to form heterocysts allows them to fix N_2_, even when oxygen levels potentially associated with *Microcystis* colonies are high. Still, the precise environmental factors driving the dynamics of diazotrophs associated with *Microcystis* colonies remain to be explored.

**Figure 4:**
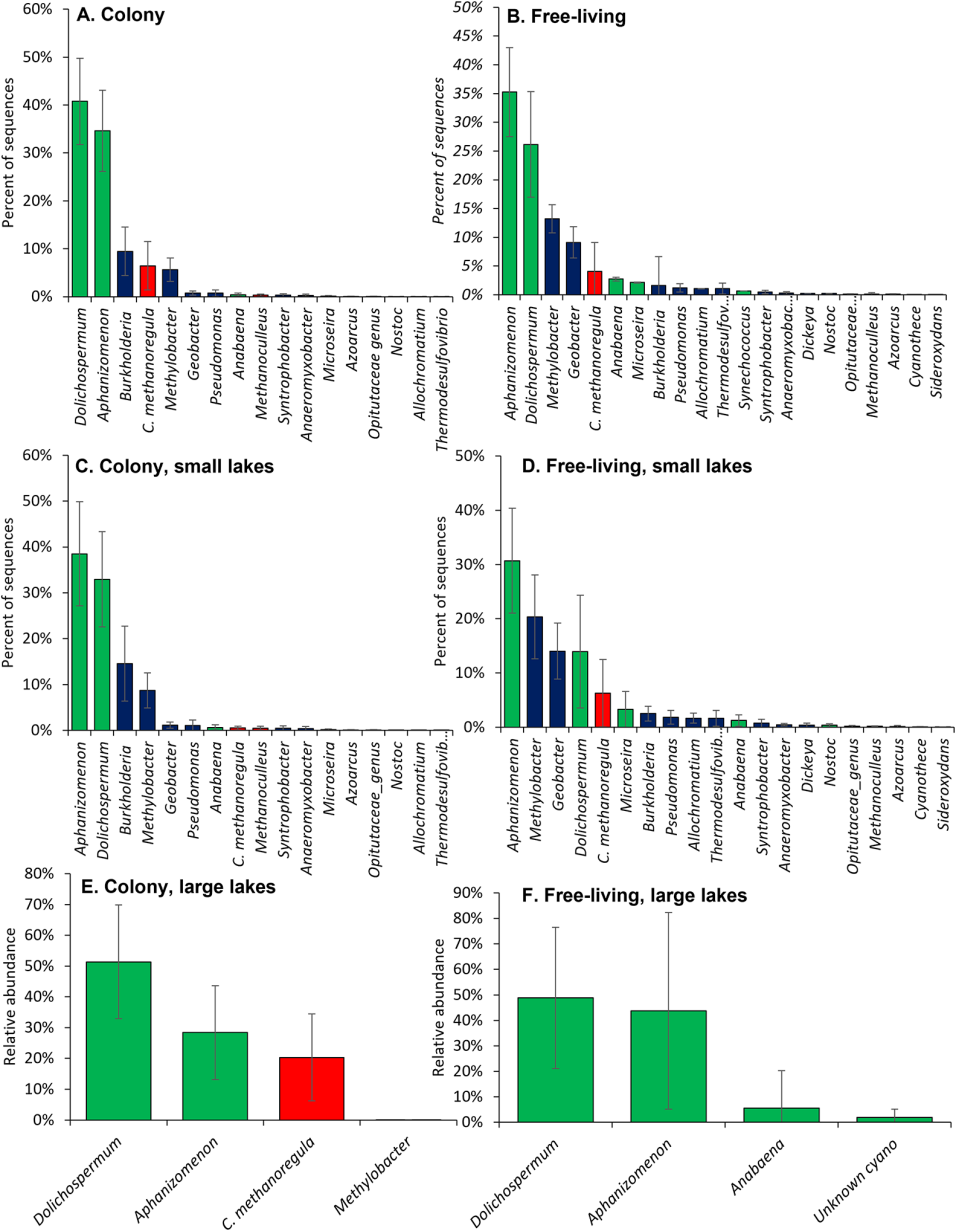
Relative abundance of *nifH*-bearing taxa for (A) all colony samples (16 taxa), (B) all free-living samples (21 taxa), (C) colony samples from smaller lakes (17 taxa; Lake Agawam and Lake in Central Park), (D) free-living samples from small lakes (20 taxa), (E) colony samples from large lakes (4 taxa; Lake Erie, Honeoye Lake, Lake Neatahwanta), F. Free-living samples from large lakes (4 taxa). Bars are means with error bars ± 1 SE. *C. methanoregula* is *Candidatus methanoregula*. “Unknown cyano” was a taxon most closely related to *Leptolyngbya.* Bars are color-coded by taxa: cyanobacteria are green, heterotrophic bacteria are blue, archaea are red.

The diversity of *nifH*-containing microbes was slightly lower in colonies, harboring fewer taxa compared to the free-living fraction (Figure 4A,B), a finding consistent with previous studies exploring 16S rRNA diversity of colonies.^17,18^ The relative abundance of noncyanobacterial taxa also differed across fractions as *Methylobacter* was the most abundant *nifH*-containing genera (12% of sequences) among the free-living fraction but were less abundant within colonies (5%; Figure 4A,B). There was a greater alpha diversity of *nifH* taxa in the smaller, more eutrophic lakes (Lake Agawam, Lake in Central Park) in terms of richness and evenness compared to the larger systems (*p* < 0.001; Kruskal–Wallis; Figures S9 and S10). For example, there were 17 and 21 different taxa present in the colony and free-living fractions, respectively, in smaller systems compared to only four taxa present in both fractions within the larger lake systems (Figure 4C–F), although several taxa (e.g., *Aphanizomenon, Methylobacter*) were represented by multiple strains of the same genus. The community composition of the smaller lakes was also significantly different from the larger lakes (PERMANOVA, *p* < 0.05; Table S4). The greater diversity of diazotrophs in the smaller, more eutrophic, systems was influenced by the presence of noncyanobacterial phylotypes present at low abundances, which may reflect higher levels of organic matter creating environmental gradients and microenvironments that allow for niche differentiation among N_2_-fixers.^60,78^ The beta diversity of the free-living *nifH* community in small lakes was significantly different from the phycosphere of the colony community from larger lakes (*p* < 0.05; PERMANOVA; Table S4), and within the smaller systems, the beta diversity of the colony and free-living fractions was marginally different (*p* = 0.08; PERMANOVA; Table S4). Despite the lower diversity of *nifH* taxa within the larger lakes, differences between colonies and free-living fractions persisted with all *nifH* sequences from the free-living fraction being cyanobacterial in these systems, whereas cyanobacterial, heterotrophic bacterial, and archaeal *nifH* sequences were present within the colony fraction (Figure 4E,F), again revealing the distinct *nifH* community associated with colonies. Among the noncyanobacteria diazotrophs, community composition varied significantly across lakes as a function of geographic distance (*p* < 0.001; Mantel test; Figure S11). Community composition of the noncyanobacteria diazotrophs within smaller, more eutrophic lakes was also significantly dissimilar from those of larger lakes (*p* < 0.001; Mantel test; Figure S12).

Compared with the combined uptake of nitrate, ammonium, and urea, N_2_ fixation rates accounted for a substantial fraction of total N uptake within the colony fraction (Figure 5). For the shallower, hypereutrophic Lake Agawam, N_2_ fixation rates were just 1% of total N uptake by the colony fraction (Figure 5). For the larger, eutrophic systems, however, N_2_ fixation was quantitatively more important, comprising 4, 7, 14, and 76% of the total N assimilation by the colony fraction in Lake Erie, Lake Neatahwanta, Honeoye Lake, and Lake Chautauqua, respectively (Figure 5). Across all systems, the importance of N_2_ fixation was varied but represented 18 ± 12% of the total N assimilated by the colony fraction, on average (Figure 5). Differences in lake trophic status and the composition of colonies may partly explain trends among lakes. For example, a greater availability of dissolved nutrients, particularly ammonium, within the most eutrophic systems would be expected to reduce N_2_ fixation rates and limit its importance.^79,80^ Supporting this, we demonstrated that ammonium additions significantly reduced N_2_ fixation rates and that N_2_ fixation was inversely correlated with ammonium assimilation. In addition, ammonium concentrations in Lake Agawam (2.5 μM; Table S1) were double those of the larger lakes (1.2 μM; Table S1) and elevated concentrations of ammonium often inhibit N_2_ fixation.^71,79,80^ These findings suggest that the importance of diazotrophy associated with *Microcystis* colonies is likely to be greatest in nonhypereutrophic ecosystems. Beyond being hypereutrophic, Lake Agawam was the only system where *Microcystis* was not the primary cyanobacteria within the colony fraction which instead was dominated by *Pseudanabaena* (76% of 16S sequences) and it was the system where N_2_ fixation accounted for the smallest percentages of N uptake (1%). This may suggest that the presence of *Microcystis* and its production of mucilage within the colonies are specifically important for supporting diazotrophic prokaryotes and dizaotrophy.

**Figure 5:**
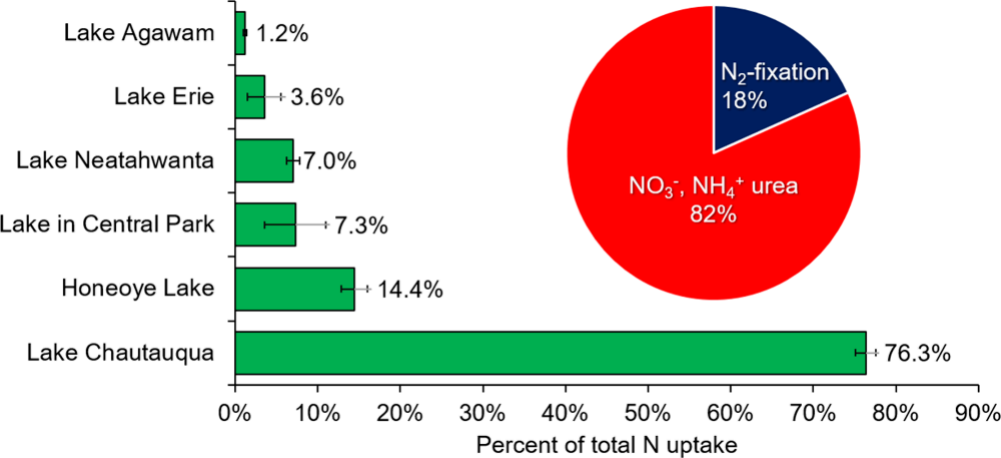
Percent of total nitrogen uptake (uptake of nitrate, ammonium, urea, and N_2_ fixation) in the colony size fraction accounted for by N_2_ fixation for each lake. Bars are means with error bars ±1 SE. Inset pie chart represents the mean for all lakes.

The existence of significant amounts of N_2_ fixation associated with *Microcystis* colonies represents an important symbiosis between *Microcystis* and co-occurring N_2_-fixing microbes, paralleling similar symbioses documented across diverse aquatic environments.^81^ Prior research has demonstrated that *Microcystis* colonies can develop anoxic microzones^82^ fostering the environmental conditions conducive for diazotrophy which must proceed in the absence of oxygen.^71,81^ By contrast, high rates of productivity associated with *Microcystis* blooms can lead to supersaturated pelagic oxygen levels,^83^ potentially inhibiting diazotrophy among free-living plankton and perhaps accounting for the lower N_2_ fixation rates within this fraction. Moreover, the abundance of organic carbon within the polysaccharide mucilage of *Microcystis* colonies provides substrate for growth and diazotrophy for heterotrophic microbes.^78,81^ N_2_ fixation associated with colonies would have clear benefits for *Microcystis* and co-occurring CHAB species given diazotrophs are known to leak excess N that is subsequently utilized by surrounding communities.^69,84–86^ For example, prior research in western Lake Erie has estimated that as much as 85% of diazotrophically fixed N is assimilated by the surrounding nondiazotrophic community.^86^

A series of studies have identified the key role N can play in controlling *Microcystis* blooms in western Lake Erie,^8,10,13,37,87^ Lake Agawam,^5^ the Lake in Central Park,^21^ and other systems around the world.^3,4^ *Microcystis* blooms often intensify when inorganic N pools are depleted and N-limitation becomes seasonally prominent.^7,12,13,87^ The extremely high biomass during blooms, particularly within the colony fraction, likely contributes toward N-limitation. Prior research has demonstrated that the peak abundances of microbes carrying nitrogenase genes in some of the lakes studied here co-occur with periods of low inorganic N concentrations.^17^ The occurrence of N_2_ fixation associated with *Microcystis* colonies may, therefore, sustain and promote CHABs under low N conditions, as N leaked by diazotrophs^70,85,86^ is assimilated by *Microcystis* blooms.

*Microcystis* blooms are an expanding ecosystem and public health threat,^2,10^ and N has been identified as a key driver of these events.^3,7,10,13,37,87^ N_2_ fixation associated with *Microcystis* colonies represented an important (on average 18%; up to 76%) source of N for supporting these CHABs, particularly in nonhypereutrophic systems. Additional research is needed to identify the precise physical relationship between diazotrophs and *Microcystis* colonies and the contribution of diazotrophically derived N to the proliferation and toxicity of *Microcystis* blooms.

## Supplementary Material



## Data Availability

All data needed to evaluate the conclusions in the paper are present in the paper and the Supporting Information. Additional data related to this paper may be requested from the authors.

## Notes

Maps of study sites (Figures S1 and S2), regression of *Microcystis* colony densities before and after colony isolation (Figure S3), relative abundance of cyanobacteria among (A) whole water, (B) free-living plankton, and (C) the *Microcystis* colony fraction (Figure S4), principal coordinates analysis showing the dissimilarity of the heterotrophic bacteria community composition between samples and among lakes (Figure S5), regression of whole water and colony fraction N_2_-fixation rates (Figure S6A), regression of total cyanobacterial chlorophyll *a* and whole water N_2_ fixation rates (Figure S6B), a time series of N_2_ fixation rates by the whole plankton, free-living and colony fractions for Lake Agawam (Figure S7A), and Lake in Central Park (Figure S7B), microscopic images of *Microcystis* colonies from the Lake in Central Park (Figure S8A), Lake Agawam (Figure S8B), Lake Erie (Figure S8C), Honeoye Lake (Figure S8D), and Neatahwanta Lake (Figure S8E), alpha diversity of *nifH* communities comparing small vs large lakes in terms of genera richness (Figure S9), alpha diversity of the *nifH*-derived communities comparing small vs large lakes in terms of the Shannon Index (Figure S10), Bray–Curtis dissimilarity of noncyanobacterial *nifH* communities as a function of geographic distances (Figure S11), Bray–Curtis dissimilarity of noncyanobacterial *nifH* communities belonging to larger and smaller lakes (Figure S12A) and communities belonging to free-living and *Microcystis* colony fractions from larger and smaller lakes (Figure S12B), environmental conditions and uptake rates across six study sites over three years (Table S1), N_2_ fixation by the colony fraction after a 4 h incubation with and without ammonium enrichment in Lake Erie, Lake Agawam, and Lake in Central Park (Table S2), δ15N content of free-living plankton and the colony fractions across the six study lakes, 2020–2022 (Table S3), and differences in the beta diversity of the *nifH* communities determined through PERMANOVA (Table S4) (PDF)
