# Supplementary material for: Nitrogen Fixation Associated with Microcystis Colonies Promotes Harmful Cyanobacterial Blooms across North American Lakes
Source: Environ Sci Technol. 2026 Jan 22;60(4):3046–56. doi: 10.1021/acs.est.5c13196 (PMC12874520; doi:10.1021/acs.est.5c13196)
Supplement: Supplementary file 1 [file es5c13196_si_001.pdf]

Nitrogen fixation associated with *Microcystis* colonies promotes harmful cyanobacterial blooms across North American lakes

Christopher J. Gobler*, Ann Marie E. Famularo-Pecora, Benjamin J. Kramer, Jennifer G. Jankowiak, Jennifer A. Goleski, Ronojoy Hem, Kendra A. Turk-Kubo, Jonathan P. Zehr

*Corresponding Author. Email: christopher.gobler@stonybrook.edu

Supplemental Information: Supplemental Figures and Tables

The Supplemental Information document is 17 pages and includes:

1. Figures S1 through S12
2. Tables S1 through S4

Supplemental Figures

Figure S1. **A**. All study sites: Lake Erie (41.823817, −83.331580), Lake Agawam (40.88148, -72.39256), the Lake in Central Park (40.77458, -73.97073), Lake Neatahwanta (43.31385, -76.42956), Honeoye Lake (42.75582, -77.50968), and Lake Chautauqua (42.113318, -79.287103). B. Inset of Honeoye Lake, C. Inset of the Lake in Central Park, D. Inset of Lake Agawam.

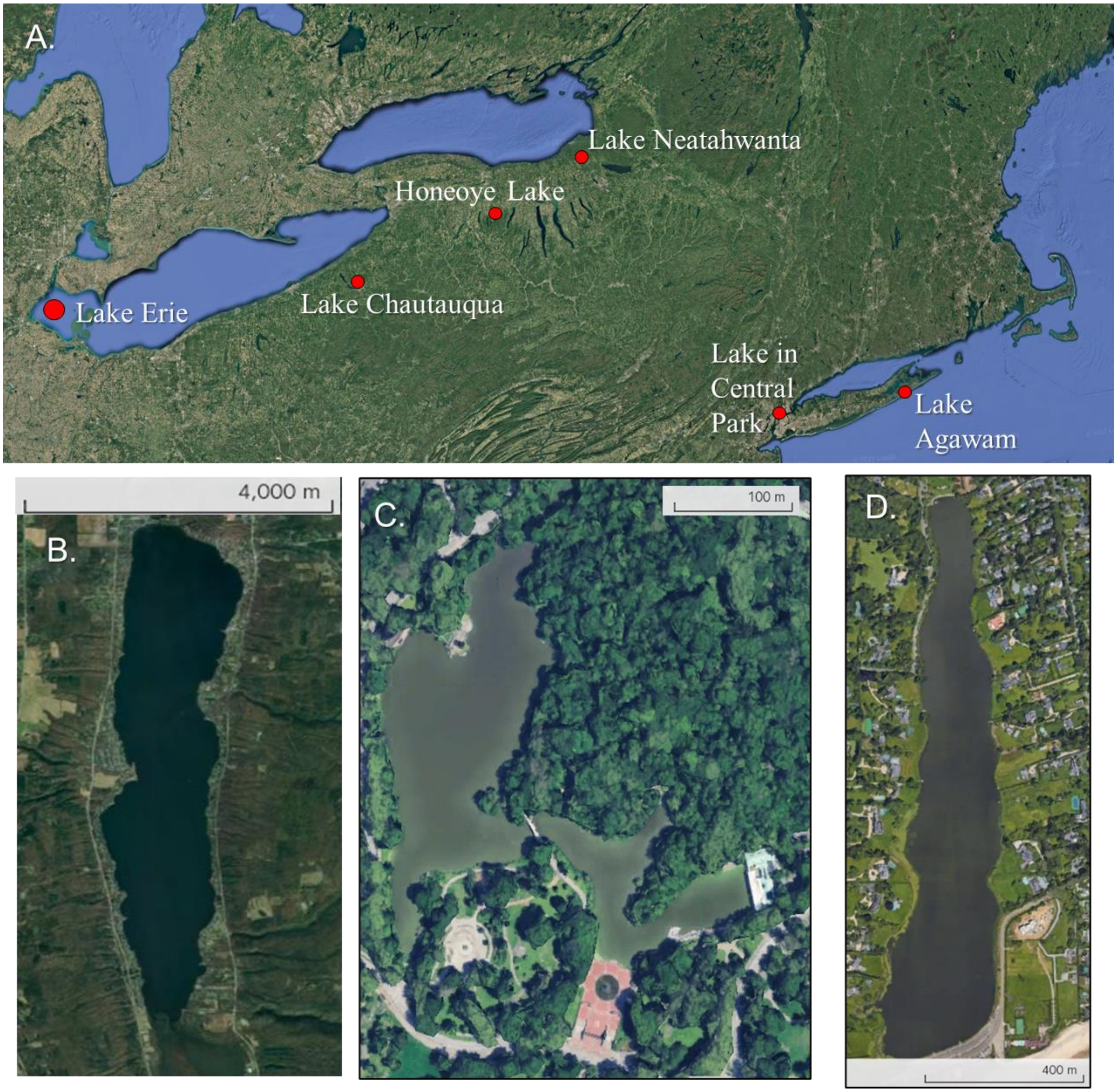

Figure S2. Inset maps of **A.** Lake Chautauqua and **B.** Lake Neatahwanta.

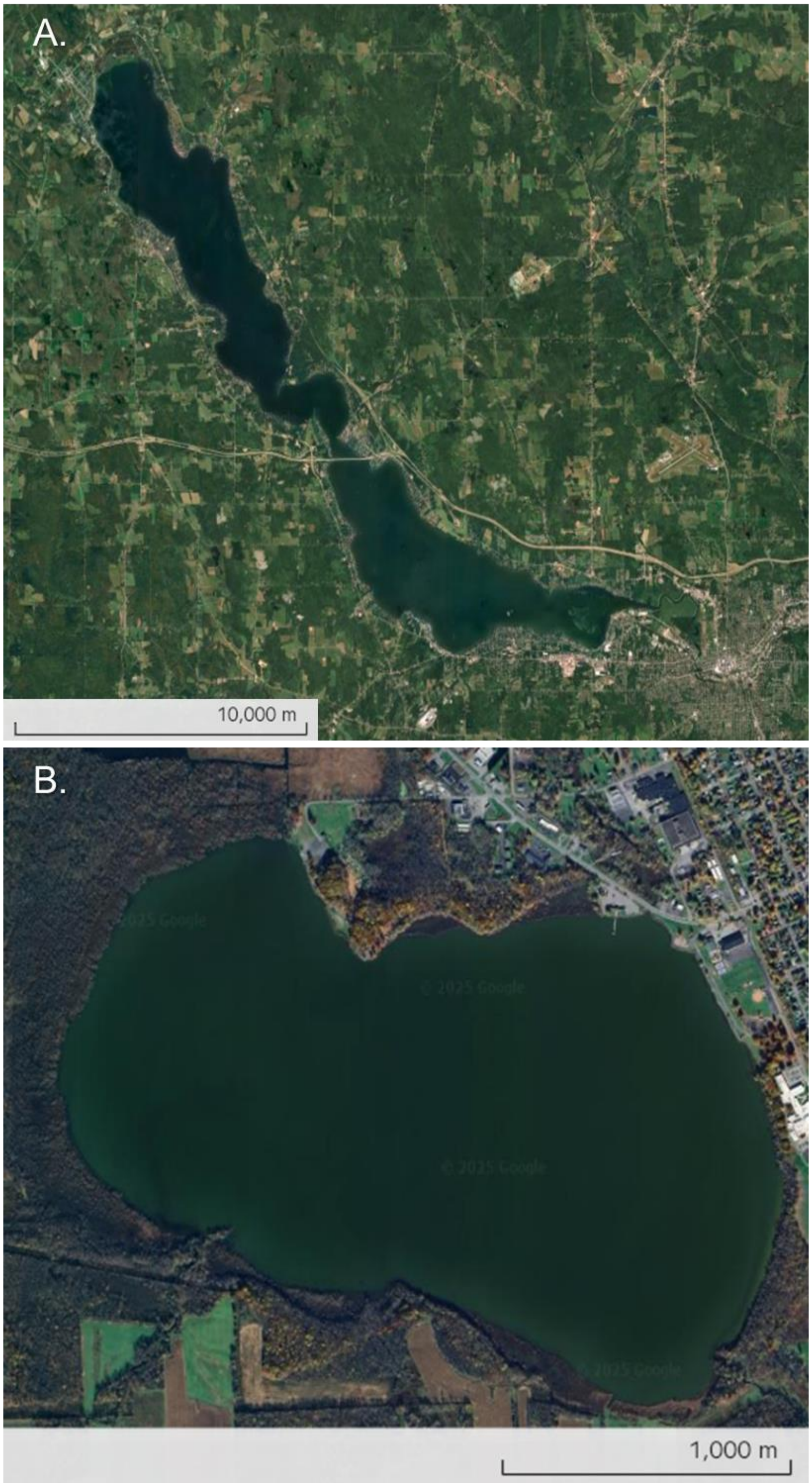

Figure S3. Density of *Microcystis* colonies in the whole water and colony fraction after the isolation process. Equation and coefficient of determination of the regression noted.

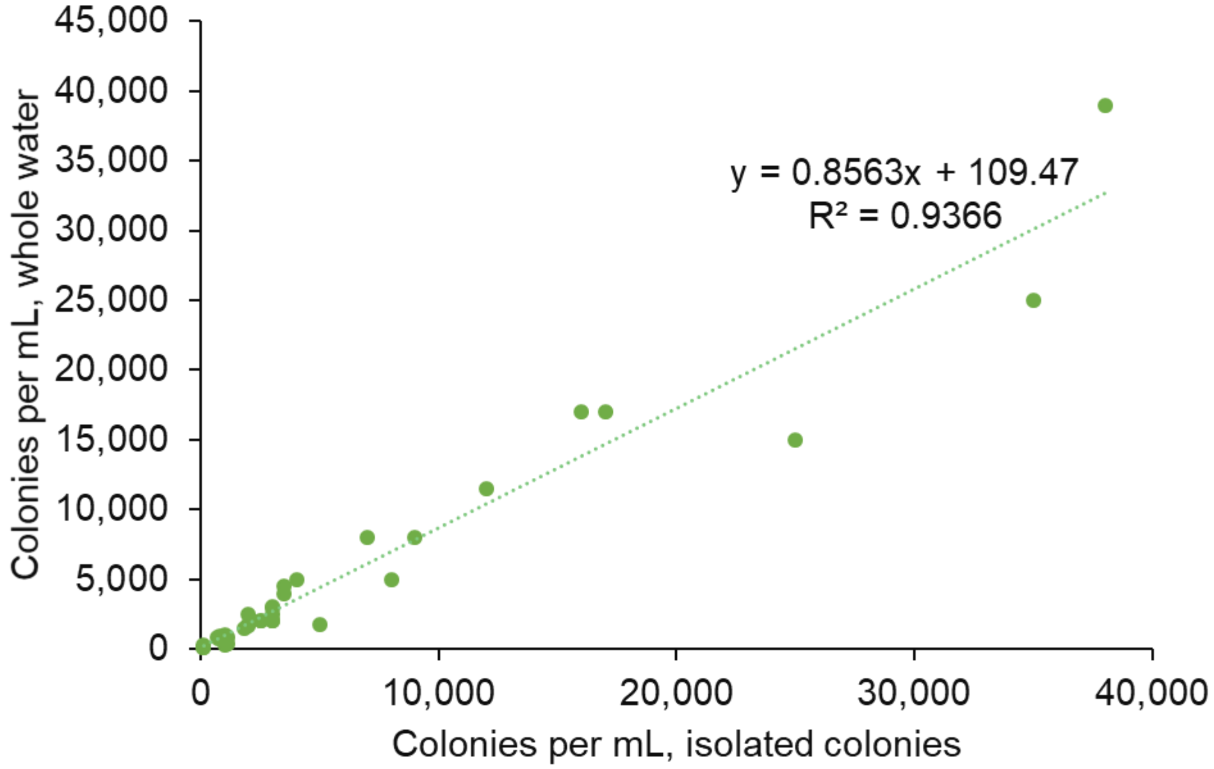

Figure S4. Relative abundance of cyanobacteria taxa identified via sequencing of the 16S rRNA gene within the A. Whole water, B. Free-living fraction, and C. *Microcystis* colony fraction.

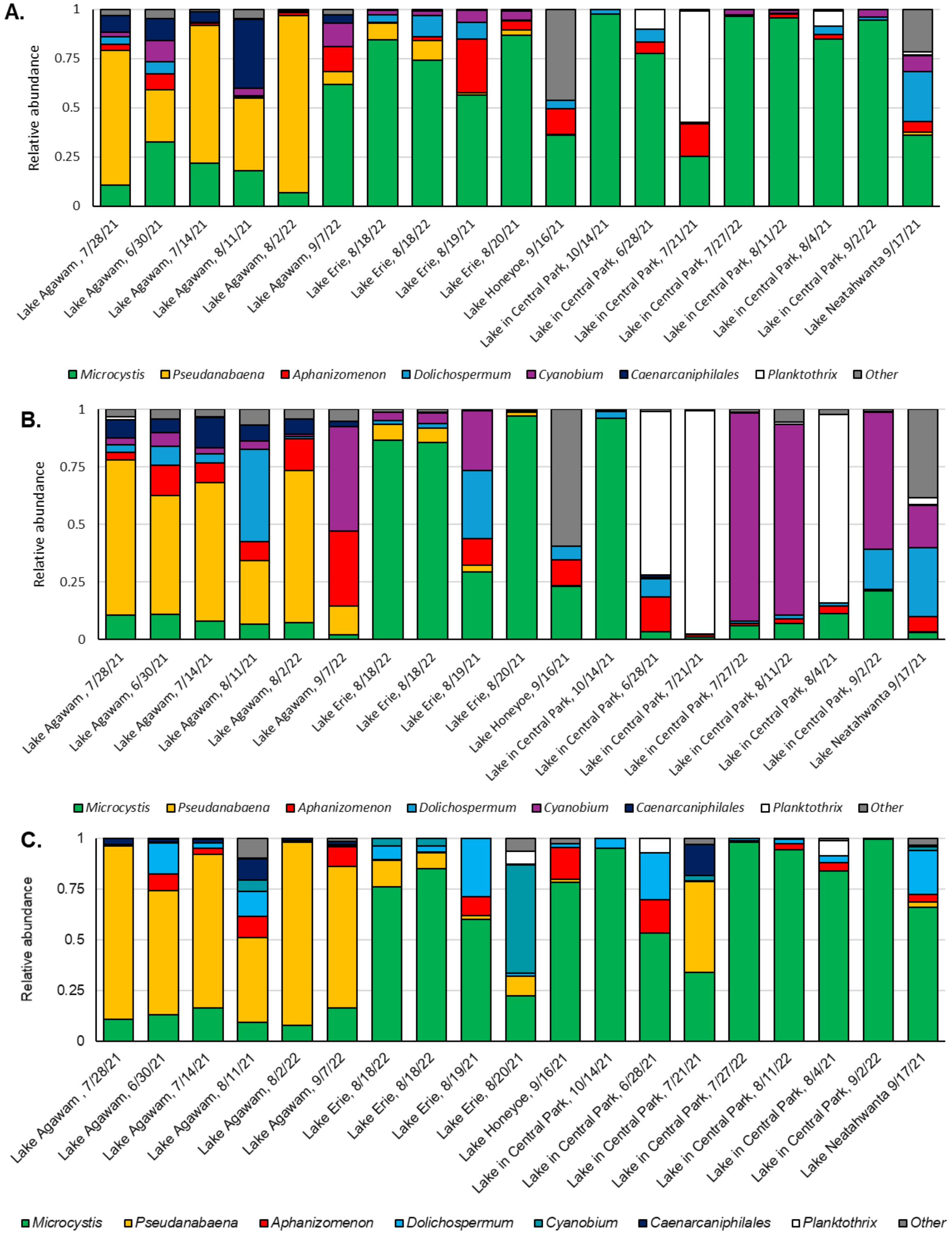

Figure S5. Principal coordinates analysis (PCoA) conducted showing the dissimilarity of the heterotrophic bacterial community compositions between samples and among lakes samples from 2021 and 2022. Colors denote the A. Plankton fractions, and B. Lake systems. Dashed circles indicate significant clustering of the communities. HE is Honeyoe Lake, LA is Lake Agawam, LE is Lake Erie, LCP is Lake in Central Park, and NT is Lake Neatahwanta.

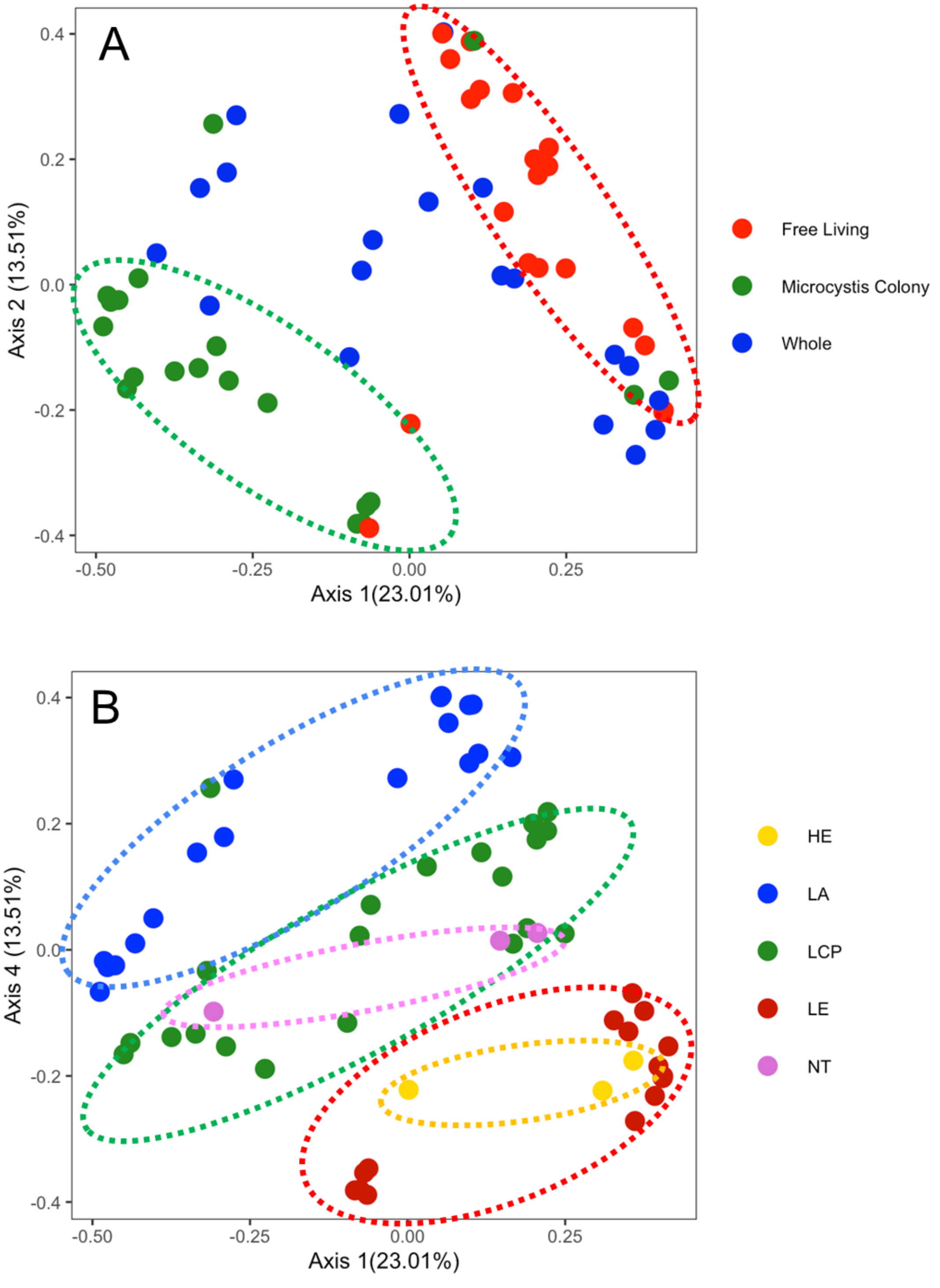

Figure S6. A. Regression of whole water and colony fraction N_2-fixation rates ($p<0.001$) and **B.** Regression of total cyanobacterial chlorophyll *a* and whole water N_2-fixation rates ($p<0.001$).

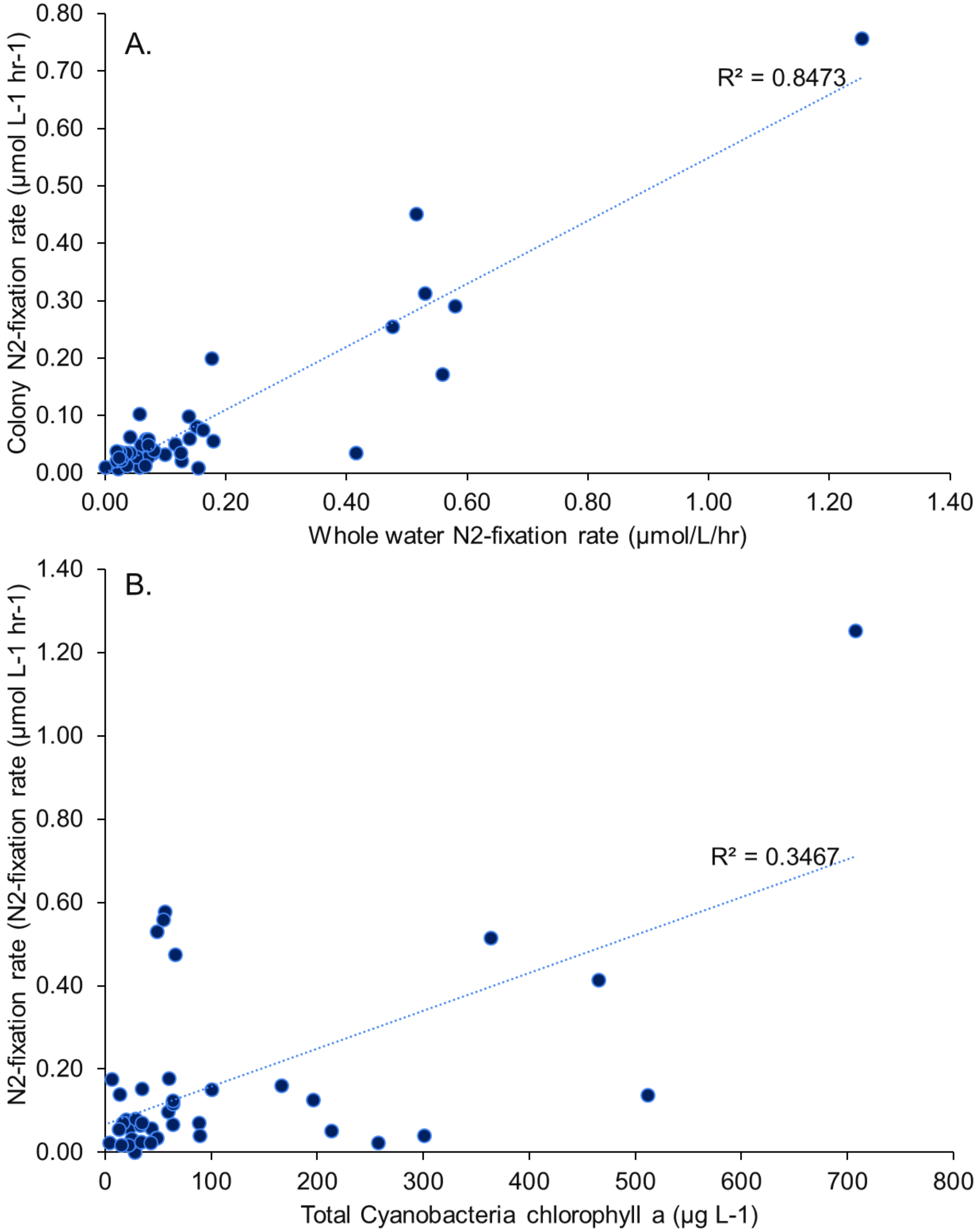

Figure S7. Time series of N_2 fixation rates by the whole plankton, free-living and colony fractions for A. Lake Agawam, and B. Lake in Central Park. Points are means with error bars are ± 1 S.E.

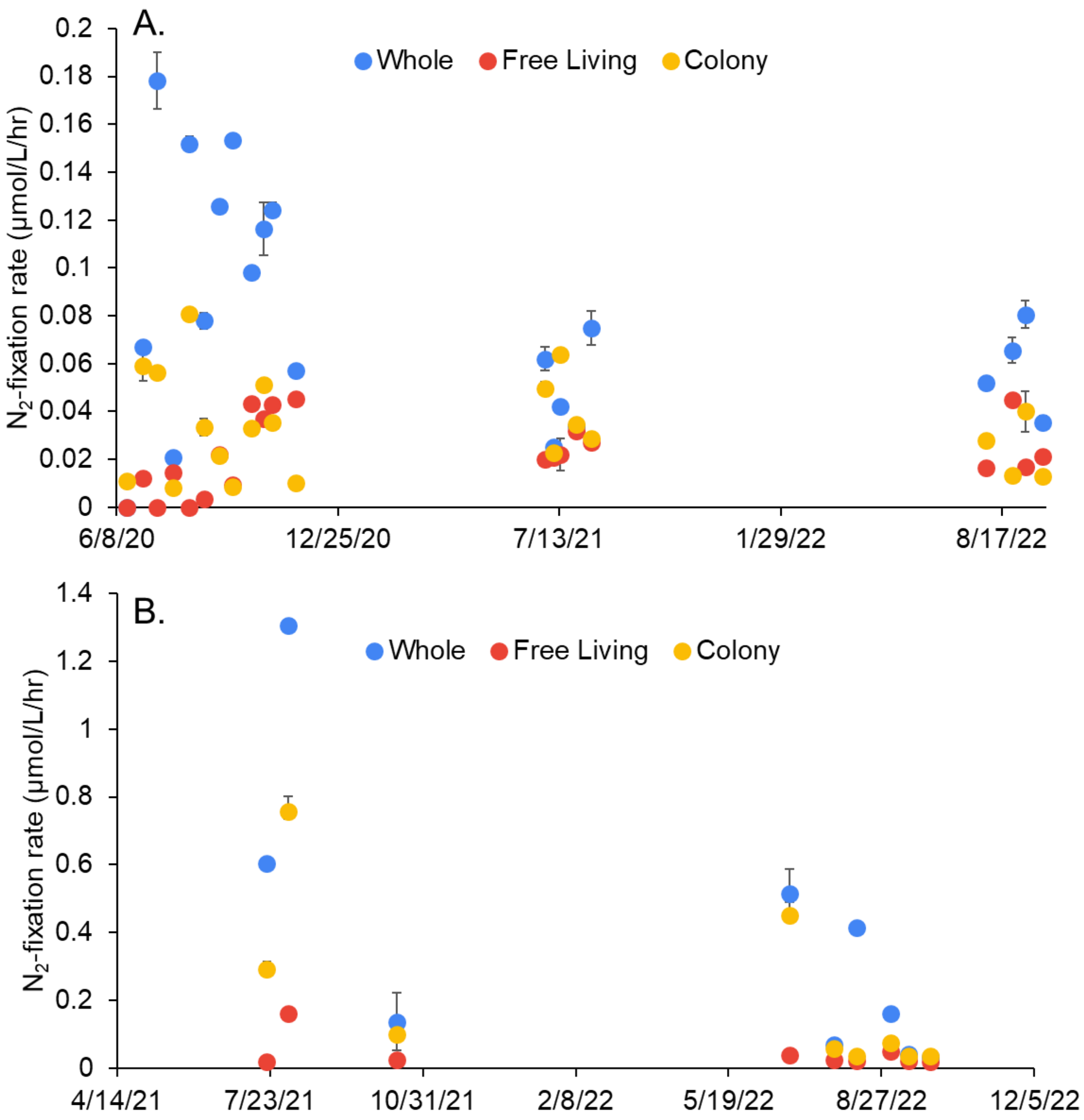

Figure S8. Microscopic images of *Microcystis* colonies from (A) the Lake in Central Park, (B) Lake Agawam, (C) Lake Erie, (D) Honeoye Lake, and (E) Neatahwanta Lake. Images from Lake Chautauqua were not available. While larger, potentially diazotrophic cyanobacteria were rarely observed in the colony fraction, the image from Honeoye Lake (D), depicts the physical intermingling of a *Microcystis* colony a filamentous cyanobacteria.

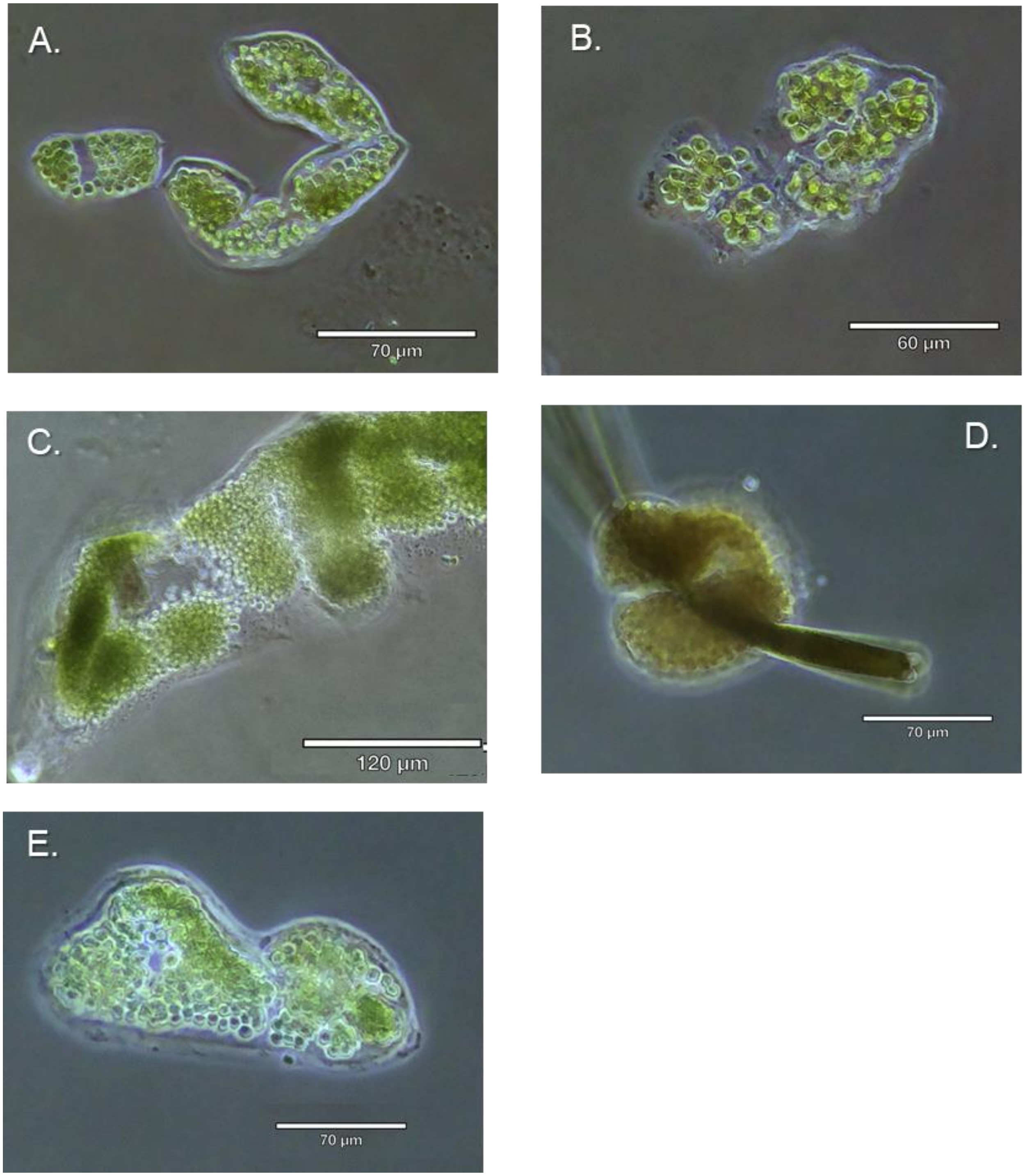

Figure S9. Alpha diversity of the *nifH* communities showing significant differences in the small vs. large lake communities in terms of genera richness determined through Kruskal-wallis analysis (p=0.000816).

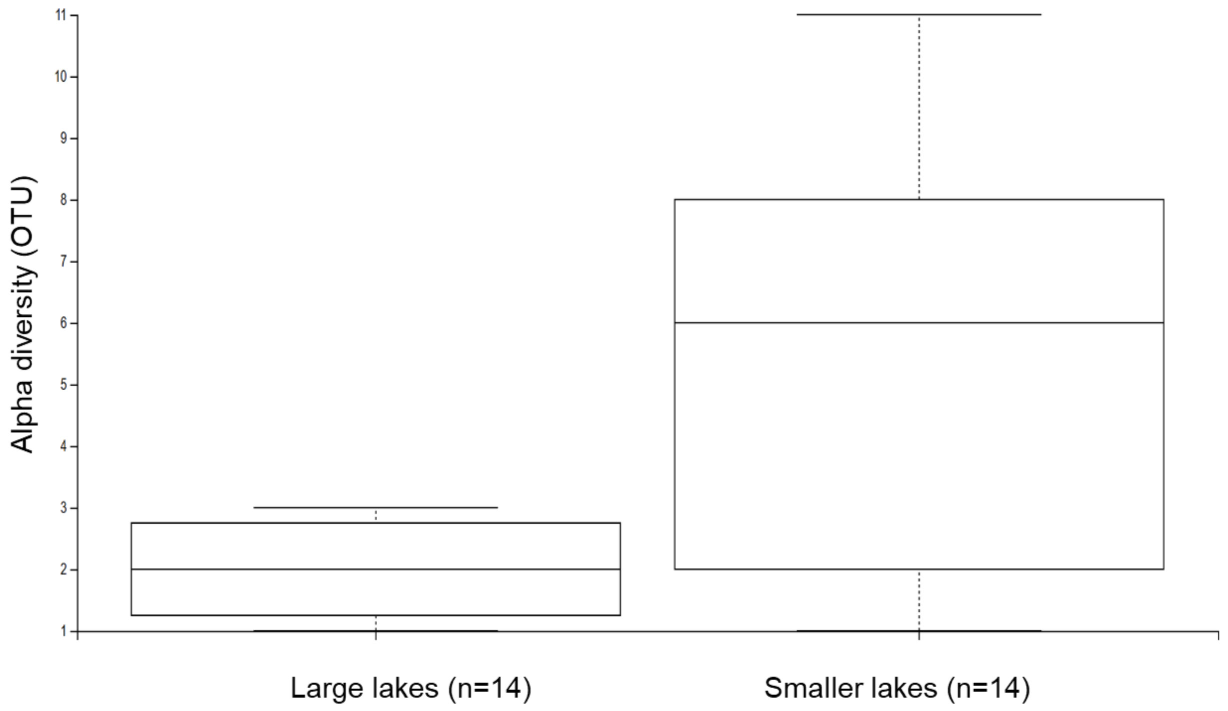

Figure S10. Alpha diversity of the *nifH* derived communities showing significant differences in the small vs large lake communities in terms of the Shannon index (genera richness and evenness) determined through Kruskal-Wallis analysis (p=0.0029).

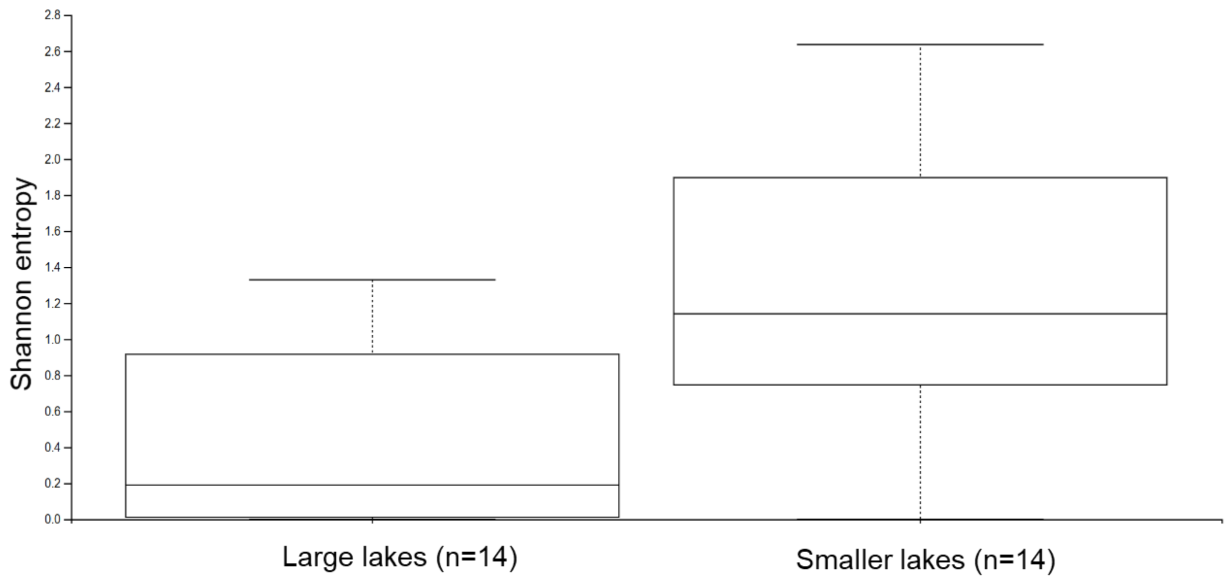

Figure S11. Mean Bray-Curtis dissimilarity levels of non-cyanobacterial *nifH* communities as a function of geographic distances (km). Mantel correlation *r* and *p* values are denoted.

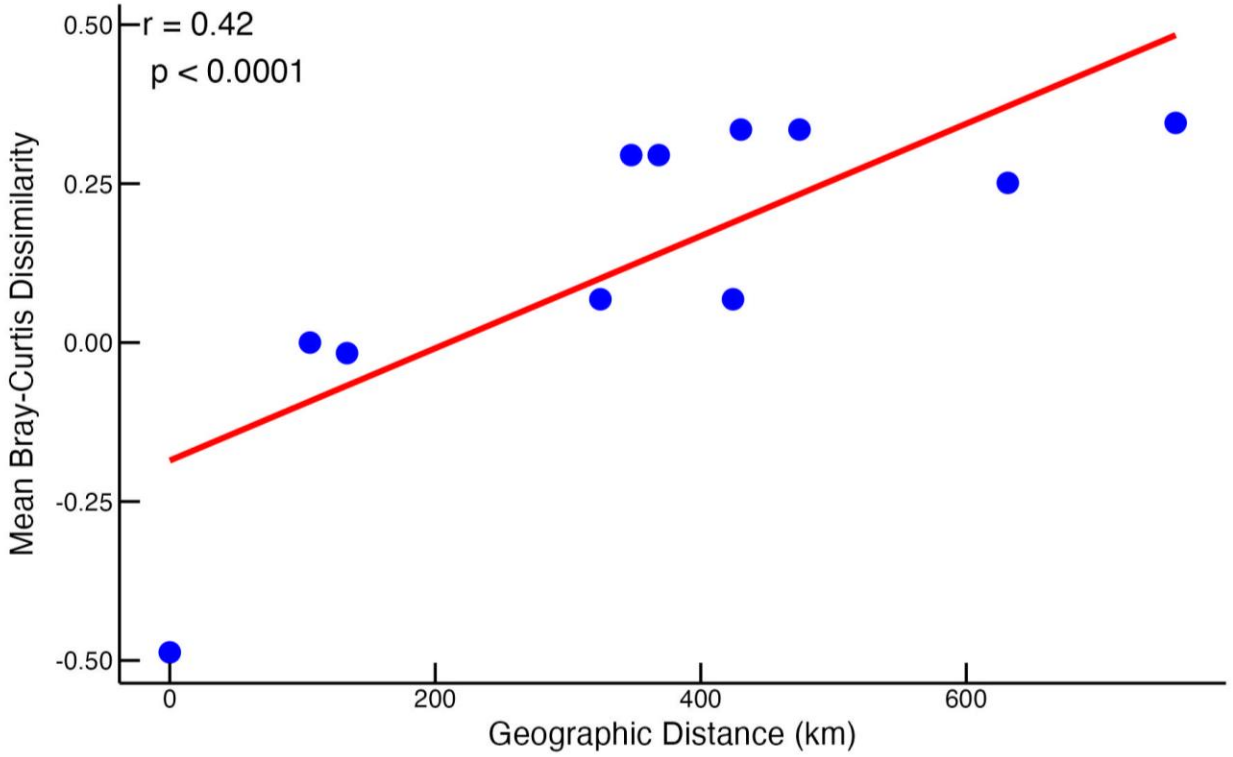

Figure S12. Bray-Curtis dissimilarity values of non-cyanobacterial *nifH* communities belonging to: A. larger and smaller lakes and B. free-living (FL) and *Microcystis* colony (MC) fractions from larger and smaller lakes. Mantel correlation *r* and *p* values are denoted.

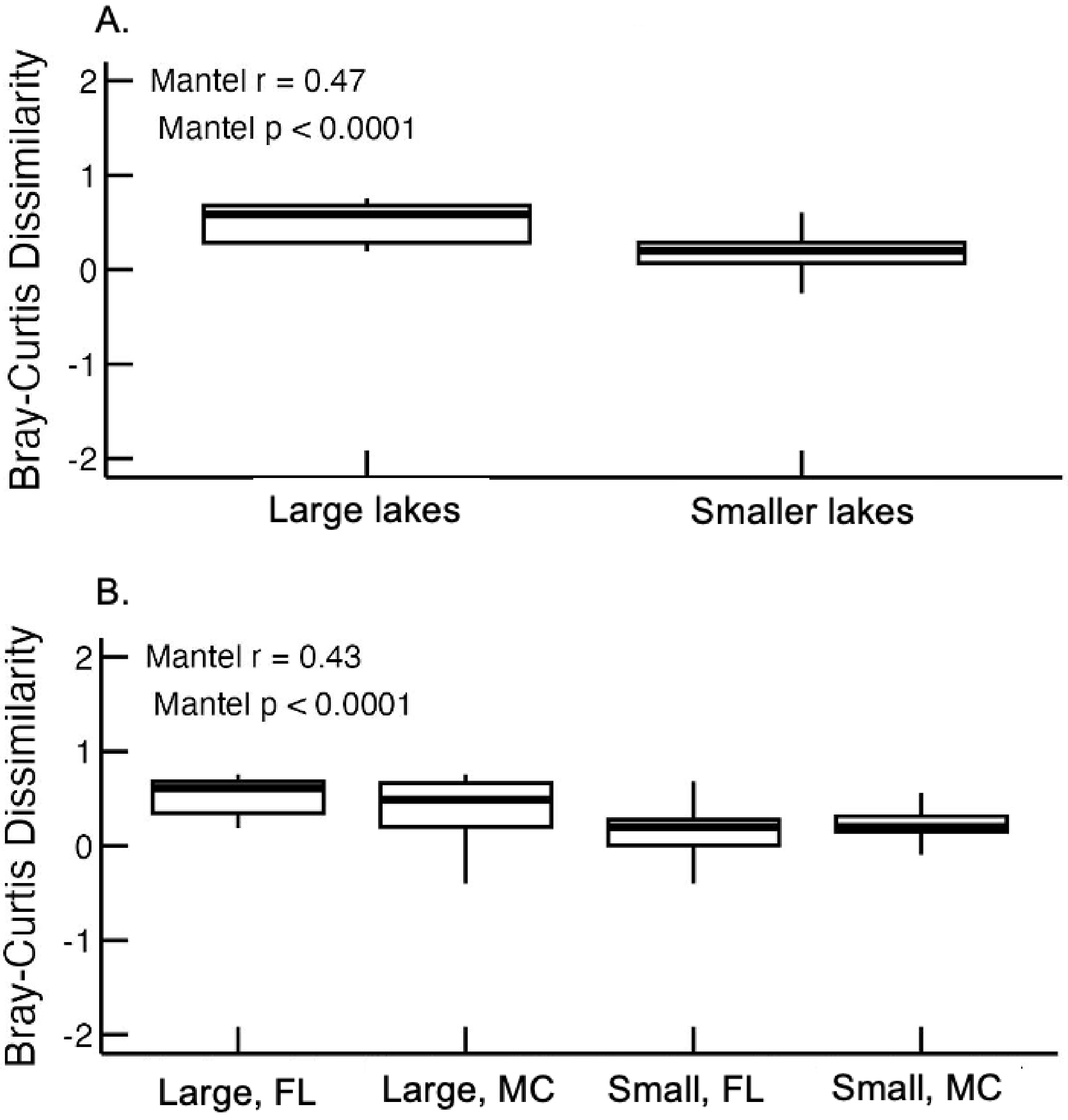

Supplemental Tables

Table S1. Environmental conditions and uptake rates across six study sites over three years. OrthoP is orthophosphate. Chla is chlorophyll *a* measured in µg L^{-1}. Cyan-chla is chlorophyll *a* attributed to cyanobacteria measured in µg L^{-1}. Microcystin is microcystin-LR equivalents measured in µg L^{-1}. N_2 fixation and dissolved N uptake are reported in µM L^{-1} h^{-1} for whole water, the free living fraction (< 20 µm) and isolated *Microcystis* colonies.

Date	Location	Temp. (°C)	Nitrate (µM)	Ammonium (µM)	Urea (µM)	OrthoP (µM)	Total chl *a*	Total cyano-chla	Percent cyano	Free living cyano-chla	Colony cyano-chla	Percent colony
6/17/2020	Lake Agawam	22.1	17.63	9.28	1.41	0.67	39	28	72%	-	27	97%
7/2/2020	Lake Agawam	27.1	0.21	0.40	1.06	6.05	67	63	95%	-	63	100%
7/15/2020	Lake Agawam	26.0	0.56	0.99	0.85	3.33	64	60	93%	-	55	91%
7/29/2020	Lake Agawam	29.9	0.67	0.59	1.11	1.67	27	23	86%	-	22	96%
8/13/2020	Lake Agawam	28.9	0.12	1.16	1.25	-	104	100	96%	-	100	100%
8/26/2020	Lake Agawam	25.1	0.81	1.65	2.43	2.72	22	20	90%	-	19	96%
9/9/2020	Lake Agawam	25.7	0.18	0.49	1.26	-	201	196	98%	-	190	97%
9/21/2020	Lake Agawam	16.2	0.55	0.63	0.64	-	37	34	92%	-	31	91%
10/8/2020	Lake Agawam	16.5	0.49	1.13	-	-	63	59	93%	-	58	99%
10/19/2020	Lake Agawam	14.9	2.32	0.68	1.20	0.73	68	64	94%	-	62	97%
10/27/2020	Lake Agawam	15.0	0.38	0.70	1.60	0.81	67	64	95%	-	62	98%
11/17/2020	Lake Agawam	13.3	10.09	11.73	1.40	0.83	48	44	91%	-	42	96%
6/30/2021	Lake Agawam	26.5	0.30	1.15	2.04	0.27	24	22	91%	1	15	69%
7/8/2021	Lake Agawam	26.3	0.00	0.71	0.96	0.17	272	257	95%	1	251	98%
7/14/2021	Lake Agawam	23.7	1.23	1.00	1.24	0.19	315	301	95%	1	274	91%
7/28/2021	Lake Agawam	27	1.30	0.89	1.07	0.21	28	24	88%	1	21	86%
8/11/2021	Lake Agawam	25.3	-	-	-	-	18	17	92%	1	16	95%
8/2/2022	Lake Agawam	24.9	1.63	9.60	2.13	3.51	222	213	96%	1	210	98%
8/26/2022	Lake Agawam	26.9	1.56	1.96	0.75	2.42	35	33	96%	1	32	97%
9/7/2022	Lake Agawam	23	3.19	3.18	1.28	0.68	30	29	95%	2	24	82%
9/23/2022	Lake Agawam	19.8	3.28	1.80	0.66	0.79	50	48	97%	2	47	98%
10/7/2020	Lake in Central Park	17.7	-	-	-	-	37	14	37%	-	13	96%
7/21/2021	Lake in Central Park	27.2	1.02	1.01	1.46	0.71	92	56	61%	10	46	82%
8/4/2021	Lake in Central Park	27.2	0.86	1.53	3.31	0.80	736	707	96%	47	613	87%
10/14/2021	Lake in Central Park	18.9	3.15	1.02	0.74	0.77	534	512	96%	9	505	99%
6/28/2022	Lake in Central Park	24.3	2.60	10.99	2.64	2.73	377	363	96%	5	298	82%
7/27/2022	Lake in Central Park	27	3.76	3.82	0.42	0.69	97	89	91%	4	86	97%
8/11/2022	Lake in Central Park	29.7	5.28	22.45	1.85	0.40	498	465	93%	9	415	89%
9/2/2022	Lake in Central Park	25.3	2.36	12.99	1.66	2.33	173	166	96%	10	141	85%
9/14/2022	Lake in Central Park	23.4	1.53	3.27	2.94	1.47	96	89	93%	3	82	92%
9/28/2022	Lake in Central Park	18.7	3.65	1.62	0.95	1.33	54	49	90%	4	42	87%
9/26/2020	Lake Chautauqua	19	0.22	0.26	0.80	1.65	51	49	95%	-	48	99%
9/29/2020	Lake Chautauqua	21	0.66	1.33	0.01	0.41	13	13	99%	-	11	88%
9/24/2020	Lake Erie	21	-	-	-	-	10	6	60%	-	7	110%
8/17/2021	Lake Erie	24.2	63.52	1.67	1.24	0.30	14	4	25%	1	3	91%
8/17/2022	Lake Erie	22.9	35.70	1.07	0.61	0.28	29	22	75%	4	18	83%
8/20/2021	Lake Erie	24.5	83.53	1.78	1.07	1.02	21	15	73%	1	6	41%
8/18/2022	Lake Erie	23	19.38	1.35	2.80	0.28	38	34	87%	11	24	73%
8/19/2022	Lake Erie	23.6	1.06	1.77	2.08	0.46	48	42	89%	8	28	66%
8/19/2021	Lake Erie	25.9	44.58	0.60	1.51	0.58	43	34	81%	6	26	75%
9/16/2021	Honeyoe Lake	22	0.34	1.40	0.69	0.27	84	66	78%	4	44	68%
9/17/2021	Lake Neatahwanta	21.6	2.17	1.01	1.30	0.31	67	54	82%	22	22	41%

Date	Location	Microcystin	Total N_2 fixation	Free living N_2 fixation	Colony N_2 fixation	Total dissolved N uptake	Free living dissolved N uptake	Colony dissolved N uptake
6/17/2020	Lake Agawam	11	0.00	0.00	0.01	18.56	5.59	5.23
7/2/2020	Lake Agawam	27	0.07	0.01	0.06	2.42	0.11	8.81
7/15/2020	Lake Agawam	35	0.18	0.00	0.06	2.62	0.14	2.46
7/29/2020	Lake Agawam	23	0.02	0.01	0.01	2.54	0.15	4.32
8/13/2020	Lake Agawam	103	0.15	0.00	0.08	2.67	0.44	4.66
8/26/2020	Lake Agawam	12	0.08	0.00	0.03	4.24	0.31	5.45
9/9/2020	Lake Agawam	94	0.13	0.02	0.02	2.95	0.06	10.15
9/21/2020	Lake Agawam	21	0.15	0.01	0.01	1.73	0.29	2.01
10/8/2020	Lake Agawam	59	0.10	0.04	0.03	1.22	0.07	1.55
10/19/2020	Lake Agawam	33	0.12	0.04	0.05	3.32	0.09	1.85
10/27/2020	Lake Agawam	49	0.12	0.04	0.04	2.68	0.11	3.88
11/17/2020	Lake Agawam	22	0.06	0.05	0.01	0.64	0.04	0.47
6/30/2021	Lake Agawam	30	0.06	0.02	0.05	-	-	-
7/8/2021	Lake Agawam	274	0.02	0.02	0.02	-	-	-
7/14/2021	Lake Agawam	244	0.04	0.02	0.06	2.05	0.07	2.37
7/28/2021	Lake Agawam	25	0.03	0.03	0.03	1.83	0.07	1.80
8/11/2021	Lake Agawam	26	0.07	0.03	0.03	-	-	-
8/2/2022	Lake Agawam	218	0.05	0.02	0.03	18.83	0.72	19.69
8/26/2022	Lake Agawam	28	0.07	0.05	0.01	-	-	-
9/7/2022	Lake Agawam	22	0.08	0.02	0.04	11.43	0.47	8.88
9/23/2022	Lake Agawam	31	0.04	0.02	0.01	-	-	-
10/7/2020	Lake in Central Park	8	0.14	0.06	0.06	-	-	-
7/21/2021	Lake in Central Park	8	0.58	0.02	0.29	2.21	0.49	0.99
8/4/2021	Lake in Central Park	150	1.25	0.15	0.76	4.82	0.22	4.83
10/14/2021	Lake in Central Park	503	0.14	0.03	0.10	3.77	0.05	1.58
6/28/2022	Lake in Central Park	127	0.51	0.04	0.45	-	-	-
7/27/2022	Lake in Central Park	35	0.07	0.02	0.06	4.12	0.51	7.90
8/11/2022	Lake in Central Park	130	0.41	0.02	0.04	9.39	0.58	6.95
9/2/2022	Lake in Central Park	45	0.16	0.05	0.08	11.94	0.32	25.25
9/14/2022	Lake in Central Park	29	0.04	0.02	0.04	-	-	-
9/28/2022	Lake in Central Park	7	0.03	0.02	0.04	-	-	-
9/26/2020	Lake Chautauqua	44	0.53	0.00	0.31	1.29	0.25	0.10
9/29/2020	Lake Chautauqua	3	0.06	0.00	0.10	0.25	0.03	0.03
9/24/2020	Lake Erie	1	0.18	0.00	0.20	-	-	-
8/17/2021	Lake Erie	2	0.02	0.02	0.04	-	-	-
8/17/2022	Lake Erie	10	0.02	0.02	0.02	-	-	-
8/20/2021	Lake Erie	-	0.02	0.02	0.04	0.21	0.04	0.40
8/18/2022	Lake Erie	11	0.03	0.02	0.02	8.41	4.10	7.63
8/19/2022	Lake Erie	8	0.02	0.01	0.03	8.07	5.25	9.81
8/19/2021	Lake Erie	-	0.07	0.03	0.05	1.62	0.35	0.96
9/16/2021	Honeyoe Lake	2	0.48	0.02	0.25	1.33	0.53	1.51
9/17/2021	Lake Neatahwanta	10	0.56	0.39	0.17	2.85	0.65	2.28

Table S2. N_2 fixation by the colony fraction after four-hour incubations with and without ammonium enrichment in Lake Erie (1 experiment), Lake Agawam (5 experiments), and Lake in Central Park (7 experiments). Mean and S.E. and *p*-value of T-tests comparing control and treatment presented.

Date	Lake	Treatment	Mean rate (µmol/L/hr)	SE	T-test p-value
8/19/2022	Lake Erie	Control	0.0340	0.0061	p<0.01
8/19/2022	Lake Erie	+Ammonium	0.0148	0.0019	
8/11/2021	Lake Agawam	Control	0.0348	0.0022	p<0.0001
8/11/2021	Lake Agawam	+Ammonium	0.0154	0.0004	
8/2/2022	Lake Agawam	Control	0.0274	0.0010	p<0.0001
8/2/2022	Lake Agawam	+Ammonium	0.0108	0.0002	
8/26/2022	Lake Agawam	Control	0.0394	0.0014	p<0.001
8/26/2022	Lake Agawam	+Ammonium	0.0245	0.0010	
9/7/2022	Lake Agawam	Control	0.0402	0.0028	p<0.01
9/7/2022	Lake Agawam	+Ammonium	0.0208	0.0017	
9/23/2022	Lake Agawam	Control	0.0287	0.0008	p<0.01
9/23/2022	Lake Agawam	+Ammonium	0.0207	0.0013	
8/4/2021	Lake in Central Park	Control	0.5106	0.0075	p<0.0001
8/4/2021	Lake in Central Park	+Ammonium	0.0220	0.0010	
10/14/2021	Lake in Central Park	Control	0.0909	0.0025	p<0.0001
10/14/2021	Lake in Central Park	+Ammonium	0.0202	0.0013	
7/27/2022	Lake in Central Park	Control	0.0708	0.0048	p<0.0001
7/27/2022	Lake in Central Park	+Ammonium	0.0291	0.0046	
8/11/2022	Lake in Central Park	Control	0.2863	0.0210	p<0.001
8/11/2022	Lake in Central Park	+Ammonium	0.0417	0.0497	
9/2/2022	Lake in Central Park	Control	0.1179	0.0052	p<0.0001
9/2/2022	Lake in Central Park	+Ammonium	0.0177	0.0017	
9/14/2022	Lake in Central Park	Control	0.0317	0.0022	p<0.001
9/14/2022	Lake in Central Park	+Ammonium	0.0159	0.0005	
9/28/2022	Lake in Central Park	Control	0.0450	0.0002	p<0.0001
9/28/2022	Lake in Central Park	+Ammonium	0.0283	0.0009	

Table S3. $\delta^{15}N$ content of free-living plankton and the colony fractions across the six study lakes, 2020 – 2022.

Date	Site	Sample Type	Mean δ15NAir (‰)	S.D.	p-value
7/29/2020	Lake Agawam	Free living	15.66	1.87	0.015
7/29/2020	Lake Agawam	Colony	9.03	0.16	
8/13/2020	Lake Agawam	Free living	13.43	0.93	0.001
8/13/2020	Lake Agawam	Colony	8.11	0.23	
8/26/2020	Lake Agawam	Free living	10.17	0.18	0.000
8/26/2020	Lake Agawam	Colony	7.77	0.35	
9/9/2020	Lake Agawam	Free living	9.38	0.40	0.008
9/9/2020	Lake Agawam	Colony	7.73	0.42	
9/21/2020	Lake Agawam	Free living	9.30	0.75	n.s.
9/21/2020	Lake Agawam	Colony	9.14	0.34	
10/8/2020	Lake Agawam	Free living	11.12	0.60	0.004
10/8/2020	Lake Agawam	Colony	8.58	0.43	
10/19/2020	Lake Agawam	Free living	9.17	0.77	0.001
10/19/2020	Lake Agawam	Colony	8.76	0.09	
10/27/2020	Lake Agawam	Free living	8.15	1.40	n.s.
10/27/2020	Lake Agawam	Colony	9.00	0.10	
11/17/2020	Lake Agawam	Free living	9.29	0.14	0.005
11/17/2020	Lake Agawam	Colony	8.70	0.12	
7/21/2021	Lake in Central Park	Free living	3.83	1.98	n.s.
7/21/2021	Lake in Central Park	Colony	4.49	0.28	
8/4/2021	Lake in Central Park	Free living	3.76	0.20	n.s.
8/4/2021	Lake in Central Park	Colony	4.19	0.08	
10/14/2021	Lake in Central Park	Free living	5.57	0.08	n.s.
10/14/2021	Lake in Central Park	Colony	5.59	0.37	
8/19/2021	Lake Erie	Free living	6.06	0.62	n.s.
8/19/2021	Lake Erie	Colony	6.47	0.15	
8/19/2021	Lake Erie	Free living	8.86	0.48	0.010
8/19/2021	Lake Erie	Colony	10.61	0.45	
8/20/2021	Lake Erie	Free living	10.11	1.24	n.s.
8/20/2021	Lake Erie	Colony	11.61	0.77	
9/16/2021	Honeoye Lake	Free living	6.56	0.46	n.s.
9/16/2021	Honeoye Lake	Colony	5.85	0.42	
9/17/2021	Lake Neatahwanta	Free living	2.36	0.19	n.s.
9/17/2021	Lake Neatahwanta	Colony	2.19	0.18	
9/26/2020	Lake Chautauqua	Free living	13.78	1.64	0.001
9/26/2020	Lake Chautauqua	Colony	4.05	0.16	
9/29/2020	Lake Chautauqua	Free living	23.14	6.13	0.012
9/29/2020	Lake Chautauqua	Colony	7.36	1.08	
7/14/2021	Lake Agawam	Free living	9.93	0.13	n.s.
7/14/2021	Lake Agawam	Colony	10.64	0.12	
7/28/2021	Lake Agawam	Free living	9.94	0.23	n.s.
7/28/2021	Lake Agawam	Colony	9.83	0.17	
6/28/2022	Lake in Central Park	Free living	5.40	0.04	0.013
6/28/2022	Lake in Central Park	Colony	4.70	0.28	
7/26/2022	Lake in Central Park	Free living	5.02	0.34	0.008
7/26/2022	Lake in Central Park	Colony	3.75	0.28	
8/11/2022	Lake in Central Park	Free living	5.81	0.61	n.s.
8/11/2022	Lake in Central Park	Colony	6.01	1.33	
9/2/2022	Lake in Central Park	Free living	4.31	0.06	n.s.
9/2/2022	Lake in Central Park	Colony	4.96	0.13	
8/2/2022	Lake Agawam	Free living	10.51	0.57	0.000
8/2/2022	Lake Agawam	Colony	10.26	0.78	
9/7/2022	Lake Agawam	Free living	9.29	0.17	0.005
9/7/2022	Lake Agawam	Colony	8.68	0.09	
8/18/2022	Lake Erie	Free living	9.98	0.15	0.001
8/18/2022	Lake Erie	Colony	9.02	0.13	
8/19/2022	Lake Erie	Free living	10.21	0.10	0.001
8/19/2022	Lake Erie	Colony	9.63	0.07	

Table S4. Significant differences in the beta diversity of the *nifH* communities determined through PERMANOVA analysis comparing fractions (free-living vs. colony), lake size (large and small) and groups (FL = free-living, MC = colony, large lake = L, small lake = S). PERMANOVAs were run using 999 permutations and psuedo-F statistics. Significant differences shown in bold and italics.

Comparison	Group 1	Group 2	Sample size	pseudo-F	p-value
Fraction	FL	MC	40	1.20	0.282
Lake Size	Large	Small	40	3.52	***0.011***
Group	FL-L	FL-S	20	3.28	***0.021***
	FL-L	MC-L	14	0.59	0.499
	FL-L	MC-S	20	1.16	0.312
	FL-S	MC-L	20	3.07	***0.018***
	FL-S	MC-S	26	1.93	0.084
	MC-L	MC-S	20	1.53	0.184
